# Innovations in Herbal Functional Beverages: From Green Formulation and Bioactivity Preservation to Sensory Optimization and Regulatory Safety

**DOI:** 10.1002/fsn3.71776

**Published:** 2026-05-08

**Authors:** Farhang Hameed Awlqadr, Syamand Ahmed Qadir, Ammar B. Altemimi, Rawaa H. Tlay, Mohammed N. Saeed, Othman Abdulrahman Mohammed, Tarek Gamal Abedelmaksoud

**Affiliations:** ^1^ Food Science and Quality Control Halabja Technical College, Sulaimani Polytechnic University Sulaymaniyah Iraq; ^2^ Medical Laboratory Techniques Department, Halabja Technical Institute, Research Center Sulaimani Polytechnic University Sulaymaniyah Iraq; ^3^ Department of Food Science, College of Agriculture University of Basrah Basrah Iraq; ^4^ Department of Food Science, Agricultural Engineering College Damascus University Damascus Syria; ^5^ Department of Nutritional Analysis and Health Kifri Technical College, Garmian Polytechnic University Kifri City Iraq; ^6^ Medical Laboratory Science Department Halabja Technical College, Sulaimani Polytechnic University Sulaymaniyah Iraq; ^7^ Food Science Department, Faculty of Agriculture Cairo University Giza Egypt

**Keywords:** bioactive stability, food processing technology, herbal functional beverages, nutraceutical formulation, personalized nutrition, sensory quality

## Abstract

The increasing consumer preference for natural health‐promoting products has fueled the development of herbal‐extract‐enriched functional beverages. These products bridge the gap between nutrition and therapy, offering bioactive compounds that contribute to antioxidant, anti‐inflammatory, antidiabetic, and gut health benefits. This review explored the integration of herbal extracts into hot and cold beverages, focusing on the challenges and innovations in food processing. Physicochemical parameters and emerging encapsulation techniques to enhance bioavailability are discussed. Sensory properties have been analyzed in recent hedonic and descriptive sensory studies, emphasizing consumer acceptance and strategies for improving palatability. Preclinical and clinical evidence has demonstrated the health benefits of widely used herbs. In addition, safety profiles, dosage recommendations, potential herb–drug interactions, and regulatory frameworks were critically examined. This review concludes with insights into future directions, including personalized herbal nutrition, AI‐assisted formulation, and sustainable ingredient sourcing, highlighting the potential of herbal beverages in functional food systems and preventive health care.

## Graphical abstract

Herbal‐extract‐enriched beverages are emerging as promising functional foods that combine sensory appeal with health‐promoting bioactives. This review summarizes their formulation in hot and cold beverages, focusing on processing challenges, physicochemical stability, sensory quality, encapsulation strategies, health benefits, safety, and regulatory considerations while outlining future trends in personalized nutrition, AI‐assisted formulation, and sustainable ingredient sourcing.

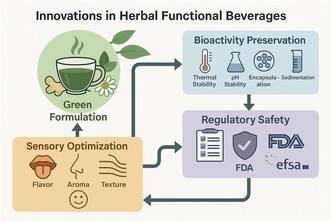

## 1 Introduction

A significant transformation has occurred in the global food and beverage industry, based on increasing consumer knowledge about health and well‐being and an increase in demand for functional drinks. One of the most attractive applications in this field is the addition of herbal extracts to beverages. Consumers are now searching for alternatives that not only hydrate or provide energy, but also have preventive and therapeutic health value. This trend is driven by evolving lifestyles and growing concerns about chronic, lifestyle‐related diseases, and the need for natural and sustainable ingredients. Functional herbal beverages cater to these needs by incorporating traditional medicinal wisdom into contemporary food technology and providing health‐giving attributes in a convenient and consumable form (Prabhu et al. 2024). Herbal extracts have found extensive applications as key constituents in the development of these beverages because they are a rich source of bioactive compounds including polyphenols, flavonoids, and essential oils. These compounds are not only powerful antioxidants and anti‐inflammatory agents but also add to the flavors, aromas, and shelf life of beverages. Medicinal and aromatic plants (MAPs), such as peppermint, rosemary, and ginger, are widely used for their multifunctional value in improving nutritional and taste potential, as well as their therapeutic properties in beverages. They are further accepted because of their natural origin, which attracts consumers looking for “clean label” products, that is, products free from synthetic additives (Sharma, Singh, et al. 2021; Sharma, Shachter, et al. 2021). As the industry becomes more established, suppliers are improving their extraction techniques to maximize the consistency and potency of their herbal ingredients. Advanced extraction methods, including steam distillation, solvent extraction, and supercritical fluid extraction, can be used to extract relevant bioactive compounds from herbs, such as sage, thyme, and hibiscus, and standardize their use in beverage formulations. This not only maintains the homogeneity of the product but also the stability of the bioactive ingredients during processing and storage (Shaw and Charters 2016). Technically corroborated according to scientific standards, herbal extracts improve the functional and taste properties of beverages. For instance, combinations of sage and wild thyme in fruit juice have been found to enhance antioxidant potential and balance flavor, which is in accordance with consumer sensory and health acceptance. This combination is usually responsible for the synergistic action, which extends the bioactive potential beyond that of single extracts (Maleš et al. 2023). Herbal beverages are being developed based on their health benefits. Certain ingredients, such as ginseng, are chosen for their adaptogenic qualities and hibiscus owing to their ability to reduce blood pressure. In addition to the antioxidant effects frequently determined by DPPH, ORAC, and FRAP assays, herbal extracts show promising metabolism‐associated health benefits, such as anti‐diabetic, anti‐obesity, and anti‐inflammatory effects. This has also been confirmed in clinical and preclinical studies, which indicate the positive effects of herbal beverage consumption on gut health, immune system activation, and oxidative stress (Maleš et al. 2022). Additionally, beverages containing plant extracts may provide technological advantages, such as long‐lasting natural antimicrobial and antioxidant activities. Herb extracts, such as cinnamon, mint, and thyme, have been shown to be effective in inhibiting microbial growth and oxidative spoilage, thus reducing dependence on synthetic preservatives. This biological preservation increases the attractiveness of the product to consumers interested in chemical‐free options (Tumbarski et al. 2023).

Meanwhile, there are some formulation hurdles to add herbal extracts into beverages, such as the thermal and pH sensitivity of bioactive compounds, incompatibility with other beverage matrix components, and the challenge of retaining physicochemical properties and sensory stability during processing and storage. Various factors affect the retention of bioactive compounds and the appearance of the resultant beverages. Sedimentation, turbidity, and taste imbalances affect consumer acceptability if they are not controlled by optimized formulations and processing methods (Lima et al. 2012). Despite these technical complications, the demand for natural beverages continues to increase. Various innovations, such as encapsulation, have been used to enhance the stability and bioavailability of herbal agents. In addition, artificial intelligence and machine learning technologies are utilized to improve formulations by providing the best level of ingredients for certain health benefits or consumer groups. Personalized beverages, which are processed according to age, sex, and health status, have been proposed as a primary trend in the next decade (Ayoub et al. 2025). Sustainability is becoming a growing focus in the development of herbal beverages. Ethical sourcing of herbs, minimal waste during processing, and switching out synthetic ingredients for those derived from plants are vital to ensuring that these beverages are not only functional, but also have environmental integrity. Adopting these approaches is beneficial for consumer confidence and the long‐term growth of the industry (Sharma, Singh, et al. 2021; Sharma, Shachter, et al. 2021). Fortification of beverages with herbal extracts is an increasingly developed area that combines traditional herbal medicine with modern nutritional science. It meets consumer needs for health‐enhancing, natural, and flavorful beverages and develops innovations that create new natural product categories.

This article reviews the main dimensions of herbal beverage development, including the selection of the extract, processing stability, sensory aspects, and health benefits, and offers a general overview of the current state of the art and future trends. This review discusses the applications of herbal extracts as ingredients in beverage products, including their selection, processing stability, sensory characteristics, and health functions. This review aimed to provide an overview of frequently used herbs and their effects on quality and consumer acceptance as well as scientific evidence to support the health benefits of these herbs. Furthermore, this review focuses on formulation difficulties, safety issues, and metrological trends in the development of functional herbal beverages.

## 2 Commonly Used Herbal Extracts in Beverage Fortification

As consumers seek more than just refreshment and functional health benefits of beverages, the application of herbal extracts in beverages has gained increasing attention. These medicinal herb extracts are rich sources of bioactive compounds such as polyphenolics, flavonoids, alkaloids, and essential oils, and exhibit antioxidant, anti‐inflammatory, antimicrobial, and adaptogenic activities. The analysis of herbs in drinks is a broad and diverse subject, covering both traditional and contemporary aspects, including the requirements of validated scientific formulations. Chamomile ( *Matricaria chamomilla* ) is one of the most commonly used herbs in beverage enrichment. An investigation of carbonated beverages enriched with chamomile extract revealed marked improvements in antioxidant properties, DPPH radical scavenging activity, and total phenolic content. The sensory attributes of the beverage were also acceptable, specifically when the extract concentration was 12%, demonstrating that chamomile imparts both health and palatability to health‐enhancing beverages (Aamir et al. 2024). Complex herbal teas, such as those composed of reed rhizomes, lotus leaves, and honeysuckle, have become popular in the market as detoxifying and cooling agents. They are typically prepared using aqueous extraction methods and are composed of compounds that promote metabolism. Studies on traditional Chinese medicine‐based beverages have shown that such mixtures can be easily mass‐produced at low cost without losing bioactivity (Shaik et al. 2023). Licorice ( *Glycyrrhiza glabra* ) and jujube ( *Ziziphus jujuba* ) extracts are commonly mixed together in order to form beverages with relaxing sweet aromas and great antioxidant activities. These extracts also showed good interactions with beverage pH and storage stability. Clinical fascination with licorice stems from its inhibition of monoamine oxidase and cholinesterase, making it a potential beverage for neuroprotection (İskit et al. 2025).

Herbs rich in polyphenols, such as mint, sage, turmeric, rosemary, and ginger, are often incorporated into functional beverages because of their diverse biological properties. These compounds are also known for their natural preservative capacities and beneficial effects on health, such as glycemic control, cardiovascular protection, and cognitive improvement. Analytical investigations have recently demonstrated the efficiency of these methods in the stability and preservation of polyphenols and their health benefits in beverage matrices (Sentkowska and Pyrzynska 2020). Some nootropic compounds derived from medicinal plants or herbal medicines, such as *Bacopa monnieri* , exhibit neuroprotective properties and improve cognitive outcomes. Considering its numerous health benefits, this plant is included in a group of functional beverages, such as whey‐based drinks and dairy alternatives enriched with plant nootropic compounds. These formulations are beneficial for brain function and have high antioxidant activity, making these compounds even more attractive in functional nutrition markets focused on improving memory and concentration (Nishanth et al. 2023). Yerba mate ( *Ilex paraguariensis* ) is used to produce probiotic fermented beverages rich in antioxidants. These drinks not only provide the same benefits as natural caffeine, but are also good for gut health, making them a great lactose‐intolerant alternative. The association between herbal bioactive compounds and probiotic cultures is a sector of increasing interest for beverage development (Correa et al. 2017; Lima et al. 2012).

**FIGURE 1:**
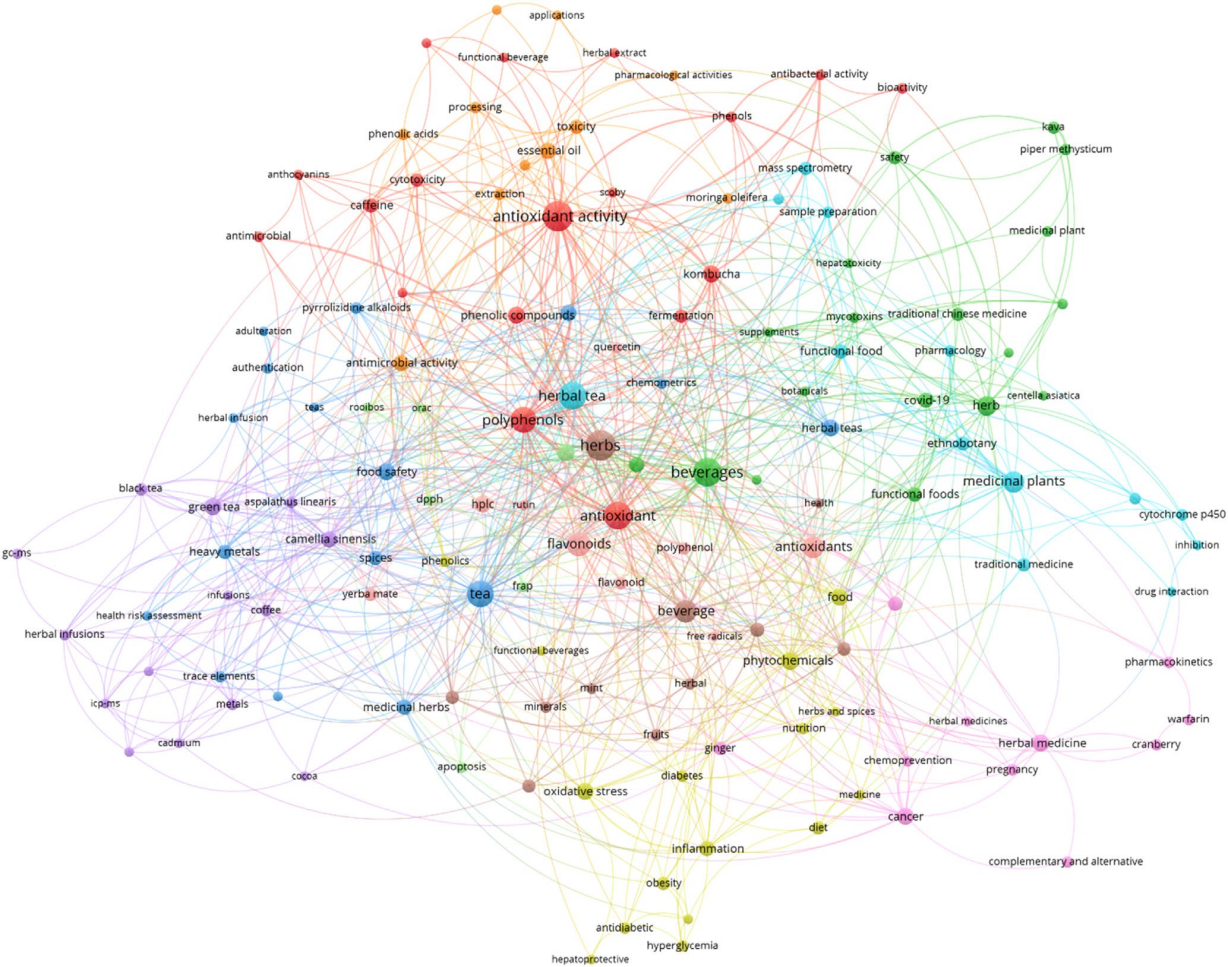
Keyword co‐occurrence map showing major research themes in herbal beverage fortification, including antioxidants, polyphenols, functional foods, and medicinal plants (VOS viewer 02.04.2025).

Ginger ( *Zingiber officinale* ) is well recognized for its anti‐inflammatory and digestive effects, and is widely used in herbal beverages. Herbal beverages containing ginger possess better sensory and nutritional properties than commercial soft drinks. The heat‐mediated bioavailability of gingerols and shogaols also justifies their use in hot and cold beverages (Semwal et al. 2015; Shahrajabian et al. 2019). Plants such as lemongrass, peppermint, sage, and holy basil have medicinal properties that have been extracted for eons in fermented beverages such as beer and wine, with no flavor‐compromising necessary. These plant add‐ons increase antioxidant power and provide distinctive taste balances, resulting in the increased use of botanicals in craft and health‐promoting alcohol items (Mascarin et al. 2023; Shiradhonkar et al. 2014). Lemonade fortification has also been examined using lindens, lemon verbena, cloves, ginger, and peppermint. These herbs enhanced antioxidant status and liking, with ginger and peppermint being the most liked by consumers. These products are refreshing and have health‐promoting qualities (Tamer et al. 2016). The multifaceted patterns of usage of herbal extracts in beverages are also demonstrated by the keyword co‐occurrence network (Figure 1). Such one‐stop drinks are easy and affordable everyday options and highlight a major health win for curcumin in a liquid diet. These drinks are designed to support post‐exercise recovery by combining electrolytes with anti‐inflammatory ingredients in a single formulation. Their formulation studies indicated the reasonable stability and consumer acceptability of the products (Yoewono et al. 2023). Other studies have investigated the use of sedative and adaptogenic herbal formulations, such as Sanghwa‐tang (a traditional Korean herbal medicine) and Fitosedan (a standardized herbal preparation), as sleep‐promoting agents in beverages. These extracts are also rich in antioxidants, have relaxing properties, and are expected to have an anti‐stress/sleep‐inducing effect, offering potential for beverages marketed for relaxation and sleep quality (Berketova et al. 2021; Park, Ochiai, et al. 2017; Park, Kim, et al. 2017). Finally, apple juice with herbal extracts (*Euphrasia* and *Prunus spinosa* ) can be enriched to enhance the antioxidant capacity and stability of apple juice. The presented recipes demonstrate the suitability of herbal extracts in fruit drinks and their potential to improve the nutritional value without negatively influencing taste (Ivanišová et al. 2010). These findings cumulatively emphasize the potential of herbal extracts in beverage development. Currently, herbal ingredients increase the parameters of functional drinks, such as cognitive support, immune boosting, antioxidant power, or digestion. With the development of extraction and stabilization technologies, application range of herbs in beverages will be expanded to more product lines and traditional practices with modern deep processing will conform to the tendency for global health food market.

## 3 Beverage Types Enriched With Herbal Extracts

### 3.1 Hot Beverages: Herbal Teas and Infusions

**FIGURE 2:**
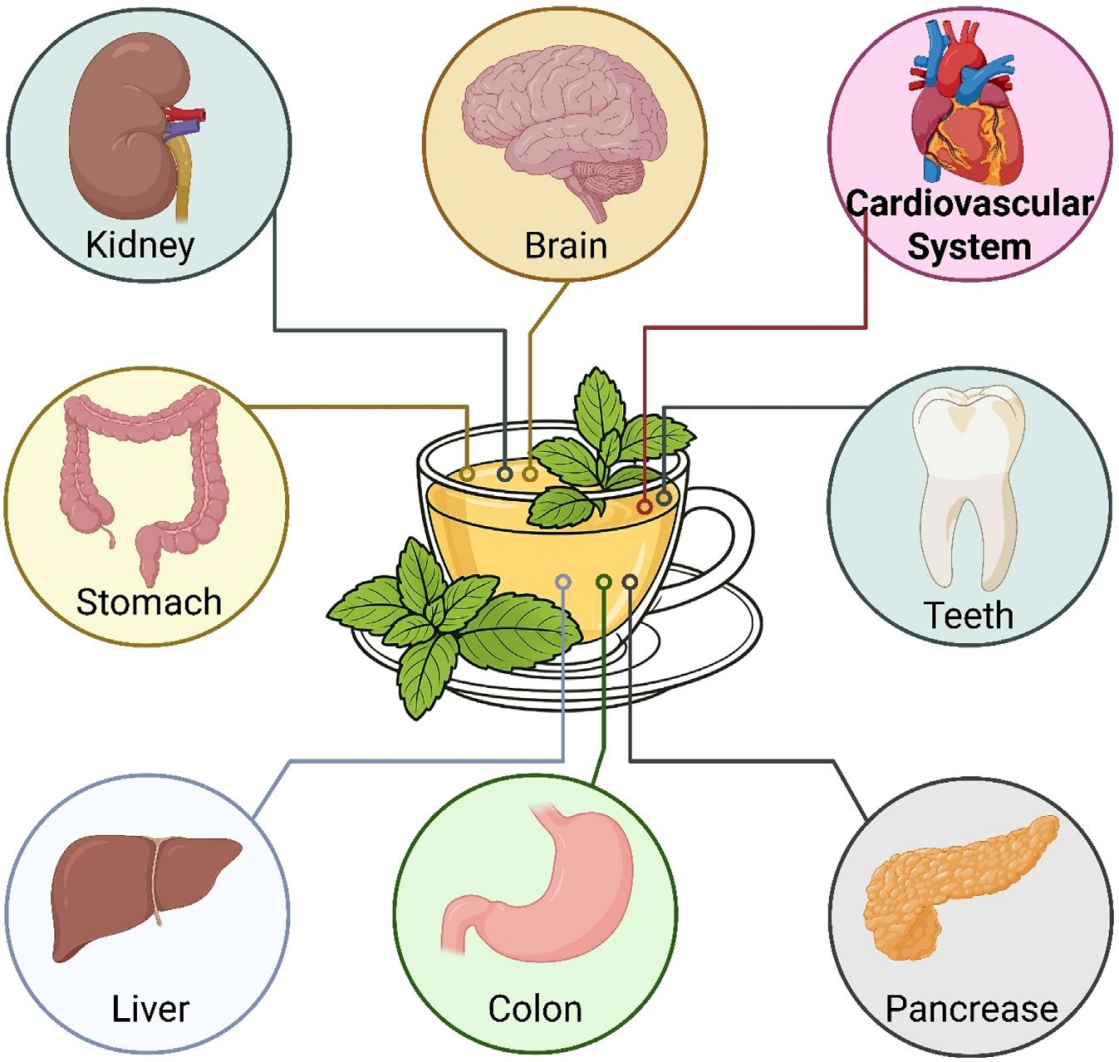
Systemic health benefits of herbal tea across major organs including the brain, kidney, liver, pancreas, and cardiovascular system, driven by its diverse bioactive compounds.

Herbal teas (also called tisanes) are non‐caffeinated beverages prepared by steeping parts of a plant, including leaves, flowers, roots, or bark, in hot water. Unlike black and green teas from *Camellia sinensis* , herbal teas contain a wide range of phytochemicals with potential therapeutic effects. Their use is growing worldwide based on perceived health benefits and cultural flavor in traditional medicine systems such as Ayurveda and Traditional Chinese Medicine. Lessons learned from scientific research show that herbal teas have antioxidant, anti‐inflammatory, antimicrobial, and even metabolic regulatory activities, all of which are provided by a high qualitative content of phenolic acids, flavonoids, terpenes, and alkaloids. These bioactivities affect several organs and physiological systems Figure 2. For instance, an extensive review found that herbal infusions such as chamomile, ginger, hibiscus, and peppermint are distinguished by high total phenolic content (TPC)—30–120 mg GAE/g and are associated with antioxidant activity (Thangavel et al. 2024). Chamomile, one of the most commonly consumed herbal infusions, has demonstrated benefits in published clinical trials that included any reduction in menstrual pain, anxiety, and postnatal sleep disturbances, likely due to the content of apigenin and bisabolol compounds that affect GABA receptors in the brain (Saadatmand et al. 2024). Similarly, the anthocyanin delphinidin‐3‐sambubioside in hibiscus tea exerts antihypertensive effects, with studies reporting reductions in systolic blood pressure (SBP) of 3–7.5 mmHg in hypertensive patients following 2–6 weeks of regular consumption (McKay et al. 2010; Patel et al. 2023). Ginger tea contains bioactive compounds, such as gingerol and shogaol, which also have anti‐inflammatory effects and are used in the treatment of nausea, stomach aches, bloating, and inflammation. A recent lipidomic analysis also demonstrated that ginger tea possesses abundant sphingolipids and phospholipids, which provide cytoprotective effects (Kumaran and Josilin 2024). Currently, peppermint tea is often used to treat abdominal discomfort and promote the muscle‐relaxing effects of menthol. Another encouraging option is birch leaf tea, which helps support the health of the urinary tract.

**FIGURE 3:**
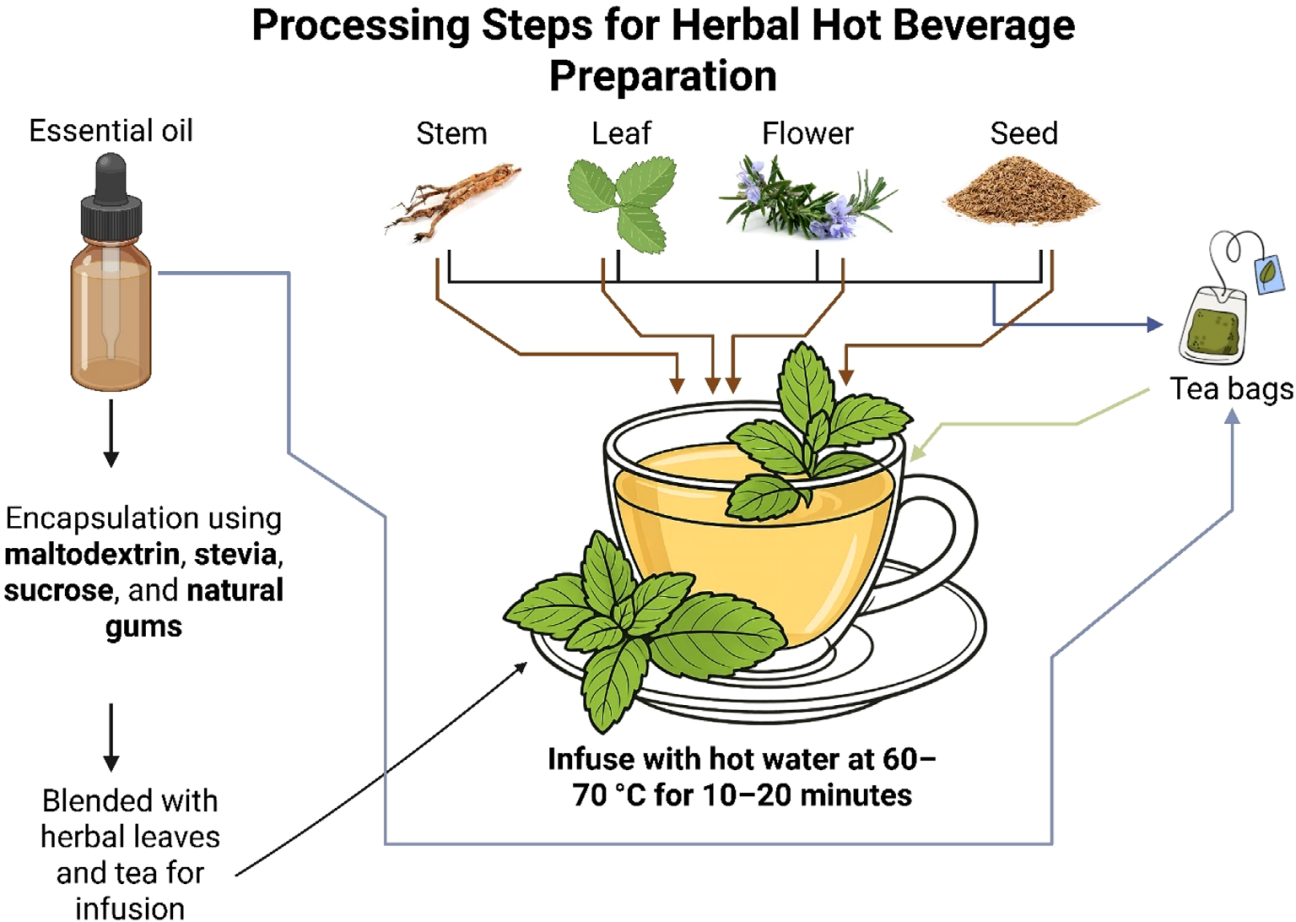
Processing steps for herbal hot beverage preparation, including infusion of plant parts and essential oils with encapsulation and tea bag formats.

**FIGURE 4:**
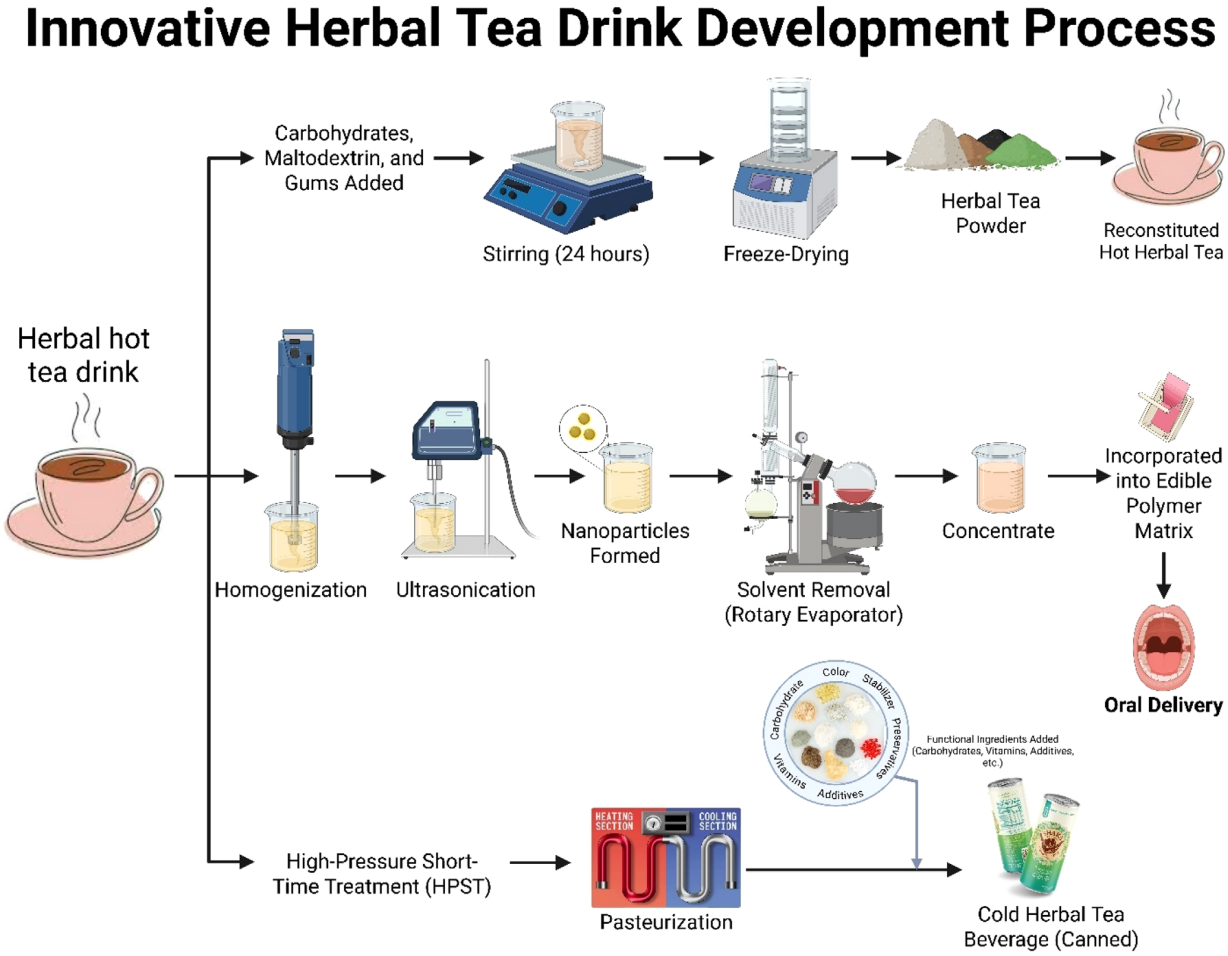
Development process of innovative herbal tea via nanotechnology, freeze‐drying, and edible matrix incorporation for enhanced stability and oral delivery.

**FIGURE 5:**
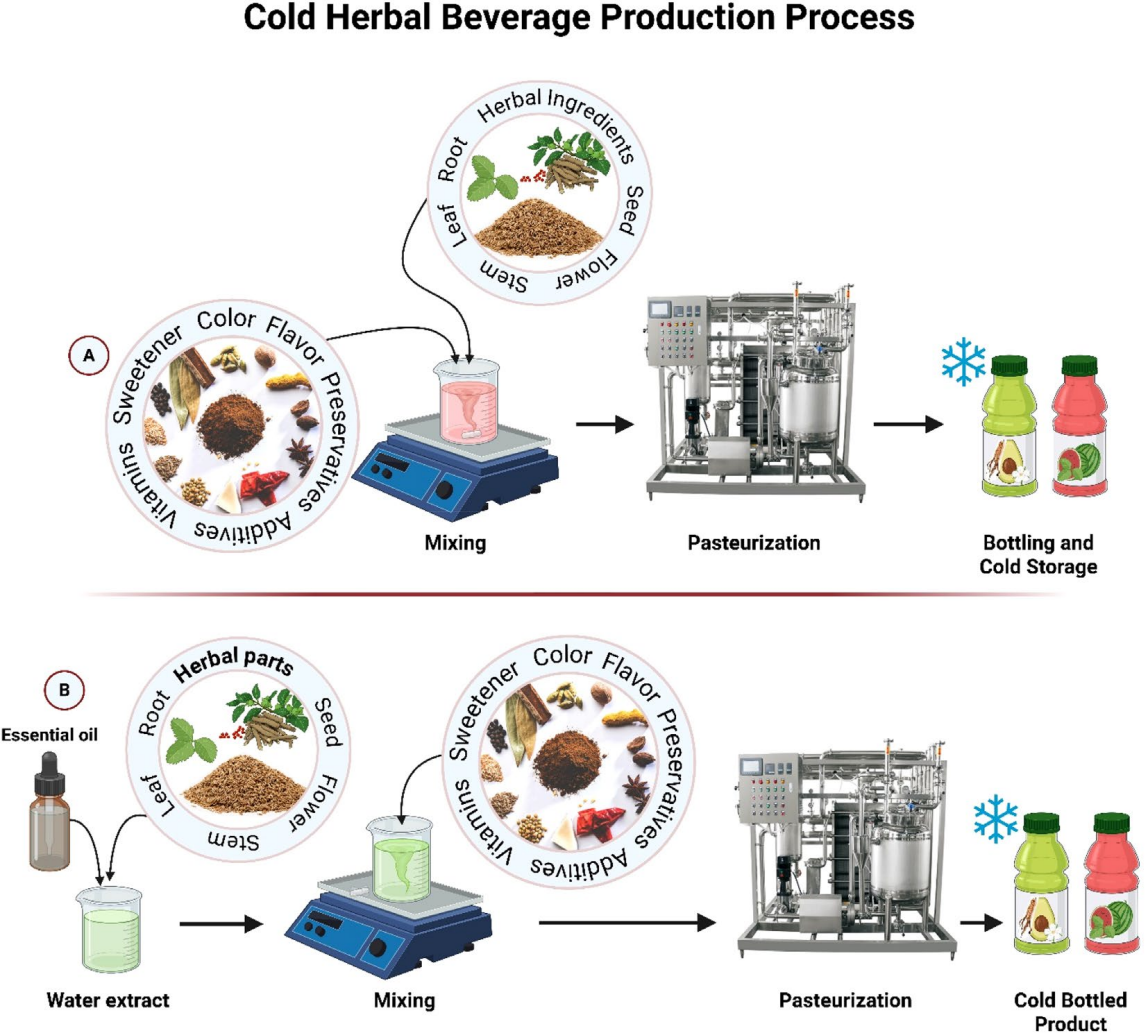
Cold herbal beverage production process: (A) Direct incorporation of herbal ingredients (leaf, root, seed, flower, stem) with flavoring agents, vitamins, preservatives, and additives followed by pasteurization and cold storage. (B) Preparation using herbal water extracts and essential oils blended with additives, then pasteurized and bottled for chilled distribution.

Popowski et al. (2024) reported that birch leaf infusions decreased inflammatory markers, such as IL‐6 and IL‐8, and prevented the adherence of *Escherichia coli* to bladder cells; therefore, it is advantageous in the treatment of urinary tract infections (Popowski et al. 2024). It is noteworthy that the processing procedures influence the configuration of bioactive compounds in herbal teas. For example, blanched and dried sharthorn leaves of *Cinnamomum porrectum* had significantly lower content of carcinogenic safrole (89% less than unblanched treatment) and higher antioxidant activities, suggesting the significance of the preparation methods in the quest for safety and effectiveness (Li et al. 2023; Sulaiman et al. 2022). The antioxidant enzyme activity was also increased, and the oxidative stress was decreased in HepG2 liver cells using white Que. Zui tea (from *Lyonia ovalifolia* buds) following treatment with ultra‐high hydrostatic pressure (Zhang et al. 2023). Although many herbal infusions are healthy, an unsafe amount of a specific ingredient is also possible owing to a lack of regulatory control (Schwalfenberg et al. 2013). One review suggested the importance of strict quality control, including identity verification, standardization and contaminant testing of unknown herbal composite products as they may contain toxic alkaloids or other accessories (Wang, Chen, et al. 2023). The standardization of plant sourcing, processing, and dosage is necessary for efficacy and safety. Studies in humans are remarkably few, and additional randomized controlled trials are needed to confirm the full therapeutic efficacy of herbal infusions. However, the present evidence clearly supports the contribution of herbal teas to the functional beverage category from an antioxidant and anti‐inflammatory perspective, and the use of such beverages as part of a daily dietary plan for preventive and therapeutic purposes. A hot liquid containing one or a combination of different herbs made for infusion aids health, including antioxidant, anti‐inflammatory, gastrointestinal, and mood effects. The range of consumption, as well as the underlying health aims to be achieved, vary with the herb and traditional use. A detailed summary of the most frequently used types of herbs, desired health effects, and recommended daily dosages is shown in Table 1. The formulation and dosage, together with the preparation of herbal hot drinks, usually involve the use of various plant parts (leaves, stems, flowers, and seeds) and essential oils, which, in many cases, are encapsulated in stabilizing substances such as maltodextrin or natural gums. These ingredients may be made into a loose mixture or tea bags and infused into hot water at appropriate temperatures. A summary of this process is shown in Figure 3. With recent technological advances, the preparation of multicomponent herbal beverages using unconventional processing techniques has become possible. An overview of the process is shown in Figure 4, where herbal hot (HH) tea was processed into powder, nano‐concentrates, and functional cold beverages via homogenization ultrasonication, nanoparticle formation, freeze‐drying, and high‐pressure short‐time (HPST) treatment. These strategies offer product diversity for improved stability and oral delivery of bioactive compounds (Figure 5).

**TABLE 1:** Commonly used herbs in beverage fortification: Health aims and recommended daily usage.

Herb type	Health aim	Usage range	References
Chamomile	Improve sleep quality, glycemic control	1–3 cups/day	Etheridge and Derbyshire (2020)
Ginger	Reduce inflammation, support digestion	1–2 cups/day	
Lemon balm	Reduce oxidative stress	1–2 cups/day	
Peppermint/Spearmint	Ease osteoarthritic stiffness, hormone balance	1–3 cups/day	
Rosehip	Relieve primary dysmenorrhea	1–2 cups/day	
Yerba mate	Increase fat oxidation, thermogenesis	500 mL/day	Maufrais et al. (2018)
Rooibos	Antioxidant capacity, polyphenol content	Variable	Damiani et al. (2019)
Hibiscus	Reduce blood pressure	1–2 cups/day	Pyrzynska and Sentkowska (2019)
St. John's Wort	Antidepressant, anti‐inflammatory	Not specified	
Lavender	Reduce anxiety, improve mood	1–2 cups/day	Poswal et al. (2019)
Holy Basil (Tulsi)	Adaptogen, stress relief	1–3 cups/day	Estiasih et al. (2025)
Kunyit Asam (Turmeric and Tamarind)	Anti‐inflammatory	Traditional drink	
Beras Kencur	Digestive tonic	Traditional drink	
Sinom	Rich in antioxidants	Traditional drink	
Wedang Jahe	Warming, cold relief	Traditional drink	
Wedang Uwuh	Immune support	Traditional drink	
Wedang Pokak	Cough and cold relief	Traditional drink	
Mint	Digestive support	Infusion	Thiagarajah et al. (2019)
Basil	Antioxidant, detoxifying	Infusion	
Lemon	Detox, vitamin C	Infused water	
Wild Thyme	Improve sensory properties, antioxidant	Juice mix	Maleš et al. (2023)
Sage	Enhance antioxidant capacity	Juice mix	
Chinese Liquorice	Cooling, reduce internal heat	Liáng chá	Liu et al. (2013)
Chrysanthemum	Eye health, fever	Liáng chá	
Prunella	Detoxifying, throat care	Liáng chá	
Alisma	Water retention, kidney health	Liáng chá	
Chinese Wolfberry	Immune support	Herbal mix	Wenli et al. (2021)
*Gardenia Jasminoides*	Cooling, anti‐inflammatory	Herbal mix	Chen et al. (2020)
Dandelion	Liver support, diuretic	Infusion	Pyrzynska and Sentkowska (2019)
Fennel	Digestive aid	Infusion	

### 3.2 Cold Beverages: Juices, Flavored Water, Smoothies, Fermented Drinks

The development and integration of herbal extracts in cold beverages have grown significantly in recent years, aligning with consumer interest in functional, health‐enhancing drinks. Herbal additives such as sage, thyme, ginger, aloe, and lemon balm are increasingly used to enrich juices, smoothies, flavored water, and fermented drinks with bioactive compounds such as polyphenols, flavonoids, and antioxidants. Recent research has demonstrated that fruit juices enriched with herbal extracts can significantly boost both the antioxidant potential and sensory appeal. One study incorporated *Salvia officinalis* (sage) and *Thymus serpylum* (wild thyme) into orange, apple, and pineapple juices. Results indicated that the orange juice with sage extract had the highest antioxidant capacity (22,925.39 ± 358.43 μM TE), while the pineapple juice enriched with wild thyme had the most harmonious flavor profile (Maleš et al. 2023). Herbal infusions have also been formulated into squash drinks, which are mainly concentrated fruit drinks with low to high total soluble solids neutralized before consumption. In another study, the incorporation of orange squash with herbal extracts enhanced consumer preference and revived ancient herbal remedies in contemporary drinks (Yadla et al. 2020). Functional beverages targeting specific health issues, including sleep, have also integrated herbal blends into cranberry and orange juices. A preference for beverages is reported, with 10%–20% content of herbal extract, which provides a balance between therapeutic effectiveness and consumer acceptability (Berketova et al. 2021). Adding to the above health benefits from the intake of herbal enriched drinks, enriched apple juice with lemon balm, oregano and salvia extracts. They demonstrated that antioxidant activities and polyphenol content were significantly enhanced when compared with those in plain juice and juice supplemented with essential oils, with lemon balm‐enriched juice showing the greatest bioactivity (Ivanišová et al. 2010). This biological enhancement not only benefits consumer health, but also appeals to the growing segment of consumers demanding natural, functional foods. Fermented drinks including kombucha and other botanically boosted beverages have also been explored. A patent outlining a composition for a hangover‐reducing beverage incorporated with fermented bean sprout juice with herbal extracts such as *Sorbus commixta* and *Hovenia dulcis* , resulting in a formulation that addresses oxidative stress and liver detoxification (Je et al. 2021; Lee et al. 2006). These beverages exemplify how cold functional drinks can serve niche therapeutic purposes. There are also technological innovations in the extraction processes used to develop these beverages. Functional drinks containing herbal extracts must balance stability, flavor, and nutritional value. The processing flow for producing stable and palatable herbal‐enriched cold beverages is shown in Figure 5. Shaw and Charters (2016) emphasized the importance of high‐quality extraction technologies for producing standardized herbal beverages and avoiding batch variability (Shaw and Charters 2016). The incorporation of extracts typically occurs during the syrup formulation stage to ensure consistent blending.

In addition, consumer palatability plays a key role in the success of commercial products. Manshi and Sharma (2023) evaluated a drink containing ginger, mint, tulsi, and lemon juices. Although the drink initially had a high antioxidant content and favorable sensory evaluation, the quality decreased significantly after 15 days at room temperature. Thus, shelf‐life optimization is a major challenge in the development of herbal beverages. In some instances, tea extracts are added to cold drinks to mask the unpleasant taste of low‐calorie sweeteners, such as stevia. These herbal additions, ranging from green to oolong and citrus teas, not only improve taste but also add subtle health benefits associated with polyphenols and tannins (Prasanth et al. 2019). This dual‐purpose use enhances the appeal among diet‐conscious consumers. This commercialization potential has led to the development of more patented herbal drinks. One example is a beverage composed of apple juice, galactooligosaccharides, inulin, and multiple herbal extracts, which offers both gut health benefits and taste optimization (Corbo et al. 2014). Other innovations include aloe‐based beverages containing herbal teas, such as chrysanthemum and mint, designed to promote digestion and improve skin health (Foster et al. 2011). These findings confirm that cold beverages, such as juices, smoothies, and fermented drinks can be significantly improved through herbal enrichment. The inclusion of herbal extracts boosts antioxidant levels, introduces therapeutic effects, and improves the consumer sensory experience. Continued research is required to optimize extraction, stability, and bioavailability, as well as to ensure consistency in production and regulatory safety. Numerous studies have explored the incorporation of herbal extracts into cold beverages to enhance their antioxidant activity, flavor, and specific health benefits. A diverse array of herbs, including sage, lemon balm, chamomile, hibiscus, ginger, and turmeric, is commonly used for this purpose. Their functional roles, dosage ranges, and applications in different beverage types are summarized in Table 2.

**TABLE 2:** Common herbal ingredients in cold beverages with their health benefits, usage ranges, and references. These include herbs like sage, mint, hibiscus, and turmeric, used to enhance flavor, antioxidant activity, and functional properties.

Herb type	Health aim	Usage range	References
Sage	Boost antioxidant activity in juices	0.5%–1.5%	Maleš et al. (2023)
Wild thyme	Improve flavor and phenolic content	0.5%–1.5%	
Lemon balm	Enhance polyphenol content in apple juice	50–150 mg/ML	Ivanišová et al. (2010)
Salvia	Increase antioxidant properties	Extract infusion	
Oregano	Boost radical scavenging activity	Extract infusion	
Chamomile	Used in squash blends for calming effect	Infused syrup	Yadla et al. (2020)
Tulsi (Holy Basil)	Enhance flavor and functional value	Juice mix	Madhuri et al. (2021)
Mint	Improve digestion, cooling effect	Juice mix	
Lemon	Vitamin C source, flavor enhancer	Juice blend	
Ginger	Anti‐inflammatory, used in herbal mix drinks	Extract in juice	
*Aloe Vera*	Digestive health and detox	Herbal water and smoothies	Foster et al. (2011)
Chrysanthemum	Cooling and antioxidant activity	Herbal tea blends	
Hibiscus	Lowers blood pressure	Cold infusion, tea	Berketova et al. (2021)
Lavender	Sleep aid, used in juice	Extract mix 10%–20%	
Thyme	Flavor and bioactivity in kombucha	Fermented tea base	Park, Ochiai, et al. (2017) and Park, Kim, et al. (2017)
Yerba Mate	Cognitive and fat oxidation boost	Cold fermented drink	Lima et al. (2012)
Green Tea	Antioxidant, stevia masking	Cold blends	Sytar et al. (2024)
Oolong Tea	Flavor enhancer, antioxidant	Cold flavored beverage	Chen et al. (2010)
Lemongrass	Relaxation and antimicrobial	Cold herbal tea	Ho et al. (2018)
*Ginkgo biloba*	Cognitive support	Herbal cold drink	
Licorice	Detox and throat soothing	Flavored syrup	Madhuri et al. (2021)
Roselle	Antihypertensive, antioxidant	Infused cold juice	Berketova et al. (2021)
Cardamom	Cooling and digestive support	Cold drink infusion	Foster et al. (2011)
Fennel	Digestive aid	Cold herb infusion	Madhuri et al. (2021)
Cinnamon	Blood sugar regulation	Infused juice	Foster et al. (2011)
Orange Peel	Zest and vitamin C	Blended in juice	Maleš et al. (2023)
Nettle	Anti‐inflammatory	Infused tea base	Ivanišová et al. (2010)
Spearmint	Cooling and flavor	Smoothies and juice	Zhang et al. (2022)
Tamarind	Refreshing, sour taste	Smoothie and sherbet	Corbo et al. (2014)
Coriander	Detox and digestive	Herbal water infusion	Berketova et al. (2021)
Basil	Anti‐inflammatory, used in squashes	Juice blend	Yadla et al. (2020)
Rose	Aromatic, mood‐lifting	Cold flower tea	Foster et al. (2011)
Ashwagandha	Stress relief	Cold blend beverage	Madhuri et al. (2021)
Curry leaves	Iron source	Green smoothie	
Moringa	Protein and iron boost	Cold mix drink	Liu et al. (2015)
Tulsi and mint	Stress relief and freshness	Cold tea blend	Madhuri et al. (2021)
Turmeric	Anti‐inflammatory	Juice or smoothie mix	Patel and Metha (2006)
Dandelion	Liver detox	Herbal extract in juice	Ivanišová et al. (2010)

Juices have been among the most popular carriers of herbal extracts. Recent developments include high‐pressure processing (HPP) for the retention of bioactive compounds and the inhibition of enzymatic browning in cloudy apple juice. For instance, the co‐application of HPP with epigallocatechin gallate (EGCG) and ferulic acid resulted in polyphenol oxidase inactivation and color stability during storage without causing loss of antioxidant ability (Marszałek et al. 2019; Tian et al. 2022). In addition, tomato juice treated with HPP showed increased bio‐accessibility of cis‐lycopene and polyphenols and improved impact on gut microbiota as compared to thermal treatment, supporting the idea that HPP conserves nutrients and exerts prebiotic‐like effects (Wang, Chen, et al. 2023). Another important invention is the provision of fruit juice compositions with improved functionality. Blends of mangoes with acerola, guava, gooseberry, and cashew apple have been reported to provide different phytochemical composition profiles and higher antioxidant properties. It is worth noting that mango‐acerola mixtures showed a positive effect by maintaining a higher vitamin C content throughout 3 months of storage (Gomez et al. 2025). These results highlight the potential of tropical fruits, not only as natural flavorings but also as sources of bioactive carotenoids and phenolics. Probiotic‐fermented products of plant origin have become popular for functional beverage innovation. Probiotic drinks based on oat‐grapes fortified with jambolan extract and fermented with *L. acidophilus* were produced. The beverages did not lose their probiotic survival at levels greater than 9 log CFU/mL over 28 days and demonstrated marked α‐glucosidase inhibition, indicating their possible antidiabetic effects. This is in accordance with the trend in the intake of herbal polyphenols, including modulation of the glycemic response and digestive health (da Cruz Nascimento et al. 2025).

Smoothies (although less established for fortification with herbs than juices) offer a high‐fiber, low‐acid medium for labile bioactive compounds. They are increasingly used to administer fat‐soluble phytonutrients, such as curcumin, which has low solubility in water and bioavailability. It is reported that curcumin could be encapsulated in orange extracellular‐derived vesicles, increasing its solubility and stability and serving as a novel carrier system in smooth and juice products (Liu, Yang, et al. 2025). Flavored water and beverages are other alternatives to herbal extracts, especially when low‐calorie functional products are being targeted. Although generally not nutrient‐dense, such drinks can be enriched with high‐purity extracts from herbs, such as mint, hibiscus, and cinnamon. Although not specifically included in the present dataset, hibiscus‐boosted flavored water was found to potentially contribute to blood pressure control and sensory refreshment (Hopkins et al. 2013; Prabhu et al. 2024). Cold‐blended juices and smoothies enriched with encapsulated polyphenols, such as chokeberries, have also been investigated. The encapsulation systems applied are based on pullulan and alginate matrices that improve the retention and release of polyphenols, as evidenced in their recently published work, which prevents oxidation, is resistant to pH changes, and can control the release in the gastrointestinal tract (Kopjar et al. 2025).

Additional technological advances include the application of atmospheric cold plasma to improve the antioxidant activities and storage stability of juices. Garimella et al. (2025) applied atmospheric cold plasma to red dragon fruit juice, and after treatment, there was a 37.59% increase in the total phenols as well as an improvement in the color and clarity. This method is a non‐thermal processing technology that inactivates target microorganisms while retaining nutritional and sensory characteristics such as flavor, texture, color, and nutritional value (Garimella et al. 2025). Sustainable ingredient procurement is also becoming increasingly important in the development of cold drinks. For example, polyphenols extracted from orange peel waste using ultrafiltration and forward osmosis have also been concentrated and reused to achieve closed‐loop processes that are in line with the circular economy principles in juice processing (Alonso‐Vázquez et al. 2025). Similarly, ellagic acid‐abundant pomegranate peel waste extracts have been optimized for health beverage purposes (Arslan Kulcan et al. 2025).

Advances in analytical and processing techniques further support innovation in cold beverage development. Untargeted metabolomics was employed to investigate the effect of the application of moderate electric fields on juice composition, which is higher in bioaccessible phenolic acids (Mercali et al. 2025). These findings will guide the optimization of processing conditions to ensure maximal nutrient delivery without loss of palatability. There is much innovation, but there are still many standardization, stability, and consumer acceptance issues that need to be overcome. Plant extracts can interact with juice matrices, causing phase separation, sedimentation, or organoleptic changes that can affect consumer acceptability. Encapsulation, pH control, and appropriate storage conditions are the best options for overcoming these limitations.

Sensory studies have highlighted the importance of balanced formulation in extract‐enriched beverages. For example, Bougainvillea‐supplemented pineapple juice also showed that balance in the formulation is important and that high levels of herbal additives would decrease the level of consumer acceptability despite its potential benefits (Mahey et al. 2025). New developments in plant‐based functional beverages have investigated the effects of different extract‐enriched juice formulations on the stability of antioxidants, microbial safety, flavor improvement, and bioavailability. These include herbal extraction, enzymatic treatment, and encapsulation technology into fruit juices, including pomegranate, orange, kiwi, and dragon fruit. The major studies describing these advances are summarized in Table 3. Rooibos herbal tea (from *Aspalathus linearis* ) increasingly gained scientific attention because of its distinct polyphenolic composition on the one hand and potential functional beverage applications on the other. Rooibos is caffeine‐free and low in tannins but relatively rich in flavonoids including aspalathin and nothofagin that are thought to be responsible for its antioxidant and anti‐inflammatory effects. Healthy and at‐risk human subjects consuming rooibos beverages ad libitum have been shown to display enhanced lipid profiles, improved antioxidant status, and decreased blood glucose levels, suggesting potential cardiometabolic benefits in functional beverage settings (Afrifa et al. 2023). In addition, in vitro studies suggest that rooibos extracts are able to protect gastrointestinal integrity and to provide beneficial support for overall gut function, highlighting its potential as a value‐added ingredient in beverage products (Pretorius and Smith 2024). Newer scientific research also reports on the modulatory effects of rooibos on redox balance and oxidative stress pathways, aspects that are pertinent to its incorporation in health‐oriented cold drink and functional beverage systems. Together, these observations justify a more thorough appreciation of rooibos in the context of herbal beverage development. In conclusion, cold beverages represent an excellent vehicle for bioactive herbal delivery systems owing to their widespread acceptability and convenience. Emerging technologies, such as HPP, encapsulation, or plasma treatment, and the use of functional ingredients such as probiotics or polyphenols, have enhanced the efficacy, safety, and sensory quality of these beverages. Further studies on extract standardization, consumer preferences, and ingredient interactions will open opportunities for cold herbal beverages to promote health and prevent.

**TABLE 3:** Overview of recent Scopus‐indexed (2025) literature articles on herbal and plant extract‐doped juices showing types of extracts, juice matrices, added materials, and purposes of health or processing.

Scopus type	Extract type	Juice type	Herb	Added materials	Aim	References
Functional dairy beverage	Radish microgreens juice	Lassi (dahi‐based)	Radish microgreens	Dahi, water	Enhance antioxidants and shelf stability	Gunjal et al. (2025)
Probiotic beverage	Jambolan ( *Syzygium cumini* )	Oat‐grape juice	Jambolan	*Lactobacillus acidophilus* , grape pulp, oat extract	Probiotic and antioxidant properties	da Cruz Nascimento et al. (2025)
Cloudy apple juice	EGCG + Ferulic acid	Cloudy apple juice	Green tea (EGCG), ferulic acid	None reported	Enzymatic browning inhibition	Tian et al. (2025)
Tomato juice	None specified	Tomato juice	None	High Hydrostatic Pressure (HHP)	Improve polyphenol bioaccessibility and gut microbiota	Wang et al. (2025)
Green tea juice	Tannase, protease, cellulase, pectinase	Green tea	*Camellia sinensis*	Enzymes	Improve taste and catechin extraction	Li et al. (2025)
Tropical fruit juice blend	None (whole juice blend)	Mango, acerola, guava, gooseberry, cashew apple	Multiple tropical fruits	None reported	Assess antioxidant potential and consumer acceptability	Gomez et al. (2025)
Chestnut rose juice	Not specified	Chestnut rose juice	Chestnut rose	DNA extraction methods	Compare DNA extraction quality and degradation extent	Ren et al. (2025)
Chokeberry juice	Chokeberry polyphenols	Chokeberry juice	Chokeberry	Alginate, pullulan, disaccharides	Improve polyphenol encapsulation	Kopjar et al. (2025)
Blended fruit & vegetable juice	None specified	Blended fruit and vegetable juice	Multiple	MEF, Shear Stress	Assess impact on metabolic profile using metabolomics	Mercali et al. (2025)
Pomegranate juice	Anthocyanin‐rich extracts	Pomegranate juice	Pomegranate	Al3+, thermal treatment	Stabilize color, reduce degradation	Idir et al. (2025)
Grape juice	Yeast extract foliar	Grape juice	Yeast ( *Saccharomyces cerevisiae* )	Volatile compound analysis (GC–MS)	Explore volatile changes due to pre‐harvest treatment	Moro et al. (2025)
Orange juice	Ultrasound and HPP treated	Orange juice	Navel orange	Ultrasound, high pressure	Preserve freshness and control browning	Chu et al. (2025)
Dragon fruit juice	Cold plasma treated	Red dragon fruit juice	Dragon fruit	Atmospheric cold plasma	Enhance antioxidant and physicochemical properties	Garimella et al. (2025)
Pineapple juice	Bougainvillea flower powder	Pineapple juice	*Bougainvillea spectabilis*	None	Improve nutritional and sensory qualities	Mahey et al. (2025)
Orange beverage	Polyphenols	Orange juice	Polyphenol blend (e.g., curcumin)	Extracellular vesicles from different oranges	Improve delivery and bioaccessibility	Liu, Liu, et al. (2025)
Orange peel extract	Phenolic compounds	By‐product extract	Orange peel	Ultrafiltration + Forward osmosis	Recover and concentrate polyphenols	Alonso‐Vázquez et al. (2025)
Apple juice	Astaxanthin from engineered callus	Tomato, carrot juice supplement	Astaxanthin (engineered biosynthesis)	Callus tissue enriched with astaxanthin	Enhance antioxidant capacity and safety	Jia et al. (2025)
Orange juice (detection platform)	Polyphenol‐modified MoS2 with tannic acid	Orange juice (sample)	Tannic acid (polyphenol)	MoS2/TA dual‐mode LFIA	Pathogen detection in orange juice	Yang et al. (2025)
Pomegranate peel extract	Ellagic acid	Not specified (extract context)	Pomegranate	Ultrafiltration, activated carbon	Enrichment of ellagic acid for nutraceuticals	Arslan Kulcan et al. (2025)
Lemon juice	Lemon juice + therapeutic plant complex	Lemon juice base for nanofiber	Lemon, green tea, *aloe vera* , black cumin	PVA nanofibers, egg shell membrane	Topical application, acne treatment	Gohar et al. (2025)
Sugarcane juice residue	Juice for enzymatic hydrolysis	Sugarcane juice (bagasse residue)	Sugarcane (Saccharum)	Potassium hydroxide, enzymes	Sugar recovery and enzymatic saccharification	Wang et al. (2025)

### 3.3 Functional Drinks: Energy Drinks, Sports Drinks, Nutraceutical Beverages

**FIGURE 6:**
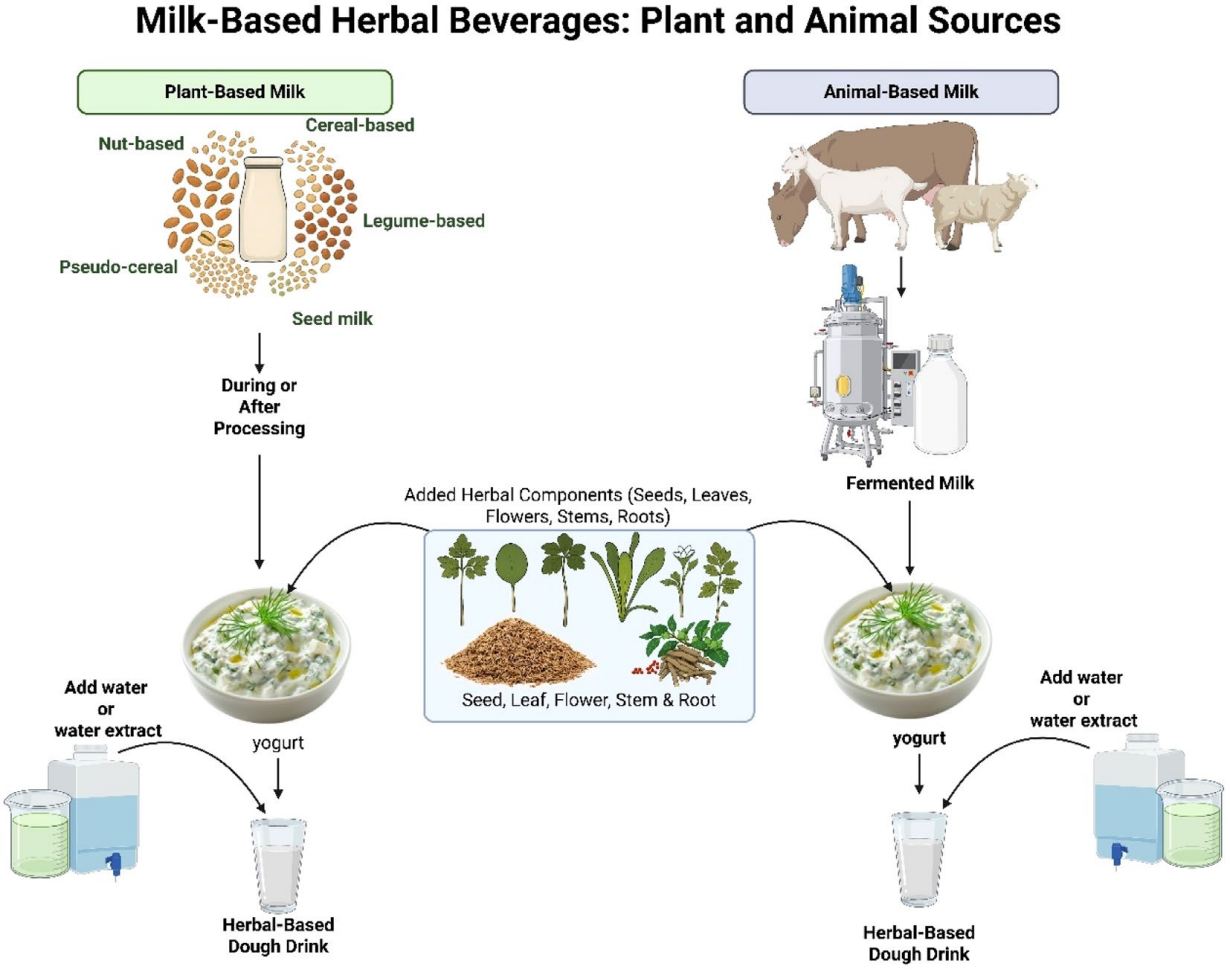
Schematic representation of milk‐based herbal beverages using both plant and animal milk sources. Herbal components (seeds, leaves, flowers, stems, roots) are incorporated during or after fermentation to produce functional doogh drinks.

The global market for functional drinks, notably energy drinks, sports beverages, and nutraceutical beverages, is growing quickly because they meet the needs of many consumers related to health, wellness, and performance. Not only do these beverages help hydration, but they also act as hosts for bioactive compounds, herbal extracts, vitamins, amino acids, and probiotics formulated specifically for energy, endurance, immunity, and recovery enhancement. This section discusses how herbal extracts and other natural ingredients play a significant role in these categories, in addition to their nutritional value and functionality shown in Table 4. The reformulation of energy drinks, traditionally high in caffeine, sugar, and synthetic additives, is emerging as a trend aimed at offering consumers products perceived as healthier. Natural substitutes for synthetic caffeine sources include green tea ( *Camellia sinensis* ), guarana ( *Paullinia cupana* ), and yerba mate ( *Ilex paraguariensis* ), all of which promote alertness; however, they also have antioxidant and anti‐inflammatory properties. A functional energy drink containing green tea extract and ginseng improves cognitive performance and attenuates oxidative stress in athletes (Wang et al. 2022). These compounds work together to promote mental concentration and exert neuroprotective effects. Ginseng beverages are gaining popularity because of their adaptogenic effects, possibly related to the *Panax ginseng* plants, which include ginsenosides reported to have an anti‐fatigue effect and endurance enhancement. Herbal functional beverages (e.g., ginseng, hibiscus, turmeric) have been indicated to play the roles of endurance, cognition, and inflammation control, and human studies suggest that athletes who consume ginseng fortified beverages report a lower perceived effort and have less of an increase in exercise recovery markers such as lactate clearance and cortisol reduction (Lee et al. 2025). These results support the inclusion of ginseng in sports and energy beverages as a viable plant‐based ergogenic aid. In addition to caffeine alternatives, sports drinks are now frequently enriched with herbal bioactives to enhance the electrolyte balance and antioxidant status. For example, beverages infused with *Hibiscus sabdariffa* extract have been found to reduce post‐exercise inflammation and oxidative stress owing to their high anthocyanin content. A double‐blind study by Olabiyi (2020) demonstrated that athletes who consumed hibiscus‐based sports drinks had lower plasma malondialdehyde levels post‐exercise, indicating reduced lipid peroxidation (Olabiyi 2020). Nutraceutical beverages, a broad category that overlaps with both energy and sports drinks, are formulated with health‐promoting ingredients that target specific health conditions such as cardiovascular support, weight management, and gut health. Probiotic‐enriched functional beverages, particularly those containing *Lactobacillus* and *Bifidobacterium* species, have shown significant benefits in modulating the gut microbiota, improving nutrient absorption, and enhancing immune function. Recent developments in symbiotic drinks combining probiotics with prebiotic herbal fibers, such as inulin or chicory root extract, have demonstrated promising results in gastrointestinal health (Pereira et al. 2023). Milk‐based herbal beverages derived from both plants (e.g., nut‐, legume‐, cereal‐, and pseudo‐cereal‐based milks) and animal sources (e.g., cow, goat) are increasingly formulated by incorporating herbal components such as seeds, leaves, stems, flowers, and roots. As shown in Figure 6, these herbs can be added during or after milk processing, particularly in fermented products, such as yogurt, to develop herbal‐based doogh drinks with enhanced functional and nutritional properties.

**TABLE 4:** Overview of functional beverages enriched with herbal ingredients, their added materials, intended health benefits, and supporting references.

Study title	Type of drink	Herbal ingredient	Added materials	Health aim	References
Green tea and ginseng‐based energy drink	Energy drink	Green tea, Ginseng	Caffeine (natural), antioxidants	Improve cognitive performance and reduce oxidative stress	Wang et al. (2022)
Ginseng‐fortified sports beverage	Sports drink	*Panax ginseng*	Electrolytes	Reduce fatigue and improve exercise recovery	Park et al. (2021)
Hibiscus‐infused sports drink for inflammation control	Sports drink	*Hibiscus sabdariffa*	Anthocyanins, Vitamin C	Reduce exercise‐induced oxidative stress	Arce‐Reynoso et al. (2023)
Probiotic functional beverage with inulin	Nutraceutical	Inulin (from chicory)	Lactobacillus spp.	Improve gut health and immunity	Zhang et al. (2023)
Curcumin‐fortified nutraceutical beverage	Nutraceutical	*Curcuma longa* (curcumin)	Piperine, encapsulants	Reduce inflammation and boost antioxidant activity	Bulbula (2021)
Beetroot juice for endurance athletes	Sports drink	*Beta vulgaris* (beetroot)	Nitrate, Natural sugars	Improve oxygen utilization and endurance	Godbole et al. (2021)
Ashwagandha‐based recovery beverage	Nutraceutical	*Withania somnifera* (Ashwagandha)	Protein blend	Enhance VO2 max and reduce stress	Deshpande et al. (2022)
Blueberry‐flavonoid drink for cognitive support	Nutraceutical	Blueberry extract	Polyphenols	Enhance cognitive function	Travica et al. (2020)
Yerba mate energy shot	Energy drink	*Ilex paraguariensis*	Caffeine, theobromine	Increase alertness and antioxidant status	Iturralde‐García et al. (2022)
Green coffee bean extract drink	Energy drink	Green coffee	Chlorogenic acids	Fat metabolism and mental alertness	Park, Ochiai, et al. (2017) and Park, Kim, et al. (2017)
Turmeric and black pepper infused beverage	Nutraceutical	*Curcuma longa* , *Piper nigrum*	Bioenhancers	Anti‐inflammatory effects	Khan et al. (2024)
Lemon balm sports drink for calming effect	Sports drink	*Melissa officinalis*	Electrolytes	Reduce stress and anxiety during physical exertion	Mathews et al. (2024)
Goji berry antioxidant drink	Nutraceutical	*Lycium barbarum*	Vitamin E, Zinc	Eye health and oxidative balance	Berisha et al. (2025)
Adaptogenic maca root drink	Nutraceutical	*Lepidium meyenii* (Maca)	B‐vitamins	Hormonal balance and vitality	Kasprzak et al. (2018)
Holy basil energy drink	Energy drink	*Ocimum sanctum*	Antioxidants	Mental clarity and adrenal support	Chaudhary et al. (2020)
Matcha‐infused recovery smoothie	Sports drink	*Camellia sinensis* (Matcha)	Protein, Almond milk	Recovery and muscle support	Zhou et al. (2024)
Amla antioxidant sports drink	Sports drink	*Phyllanthus emblica*	Vitamin C, Electrolytes	Immunity and oxidative stress protection	Pathak et al. (2024)
Reishi mushroom‐based beverage	Nutraceutical	Ganoderma lucidum	Amino acids, Minerals	Immune modulation and anti‐fatigue	Plosca et al. (2025)
Chamomile‐functional drink for athletes	Nutraceutical	*Matricaria chamomilla*	Magnesium, L‐theanine	Anti‐inflammatory and sleep support	Koshovyi et al. (2024)
Coconut water + turmeric fitness drink	Sports drink	Turmeric, coconut water	Electrolytes	Hydration and anti‐inflammatory	Kalman et al. (2012)

**FIGURE 7:**
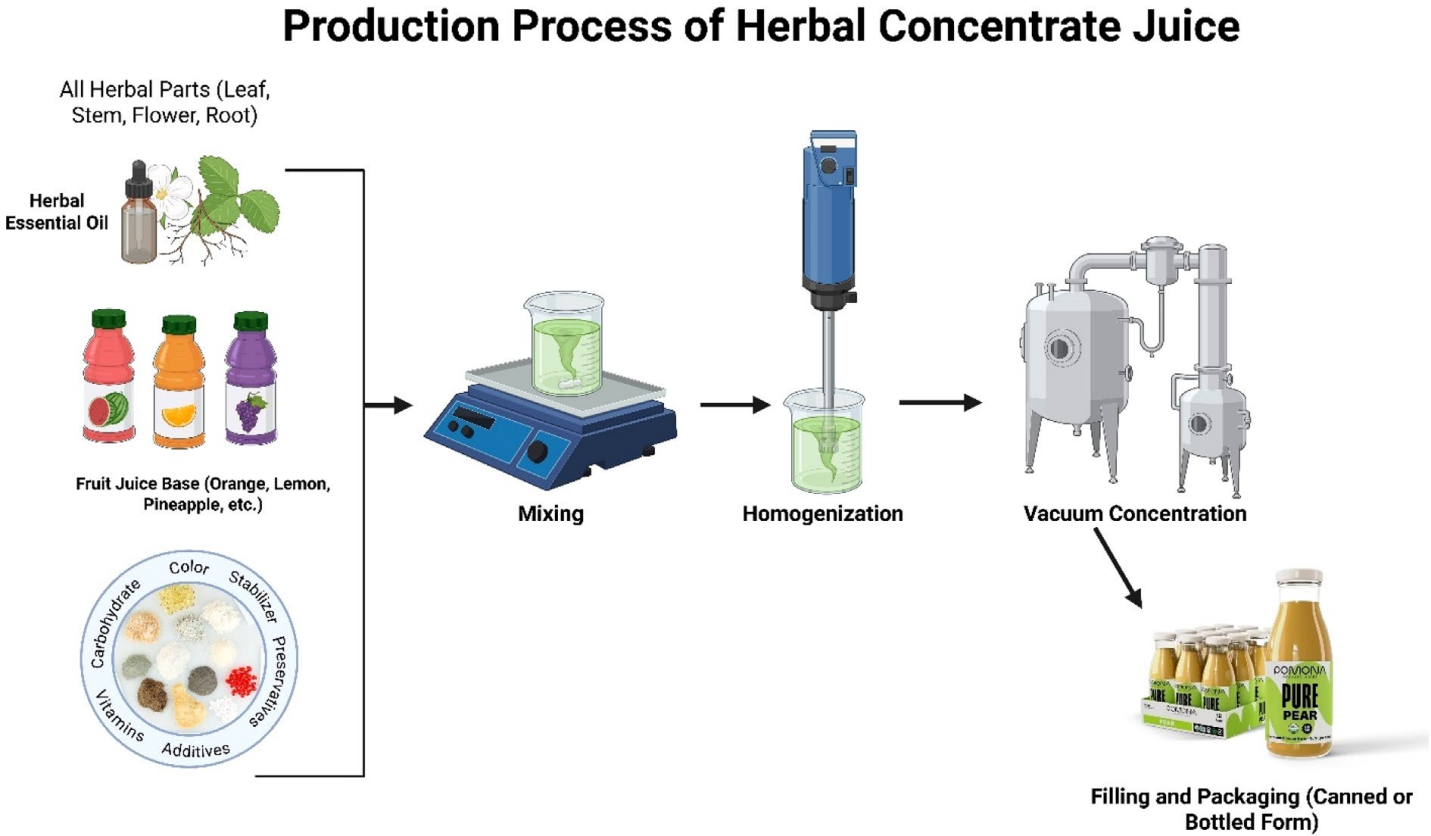
Production process of herbal concentrate juice involving essential oils, juice bases, and additives, followed by mixing, homogenization, vacuum concentration, and final packaging.

Turmeric ( *Curcuma longa* ), known for its curcuminoids, is another botanical that is gaining traction in functional beverage design. The poor bioavailability of curcumin can be mitigated by using encapsulated forms or by combining it with piperine (from black pepper), enabling its effective inclusion in nutraceutical drinks. It is shown that curcumin‐fortified beverages improved the systemic antioxidant capacity and reduced inflammatory biomarkers, such as TNF‐α and IL‐6, in moderately active adults. Another innovative approach involves the use of beetroot juice ( *Beta vulgaris* ) in sports beverages because of its high nitrate content, which promotes NO production and vasodilation. This effect has been linked to improved oxygen utilization and endurance performance (Roth 2015). Beetroot juice significantly increased the time to exhaustion during submaximal exercise across multiple trials, making it an ideal component of performance‐focused drinks (Park et al. 2021). Ashwagandha ( *Withania somnifera* ) has also been established as a popular adaptogen in functional beverages. Ashwagandha root extract, a bioactive compound that has stress evasion and anabolic effects, can help increase VO_2_ max and muscle strength when consumed in a beverage over an 8‐ to 12‐week period (Deshpande et al. 2022). Its potential to balance cortisol and provide mitochondrial support could make it trend with the increasing consumer interest in recovery and resilience beverages. Flavonoid‐containing fruits and extracts, such as blueberries, cranberries, and pomegranates, are added to functional beverages for their vascular and cognitive effects. These polyphenol sources are also known to promote endothelial function and memory retention, making them useful drinks for both energy and cognitive function. For example, it is reported that improvements in cognitive flexibility associated with blueberry polyphenol consumption and reduced mental fatigue during the completion of a cognitive task (Travica et al. 2020). Functional products offer great potential; however, issues of taste masking, bioactive stability, and regulatory hurdles remain to be overcome. Many other encapsulation technologies and natural emulsifiers, such as lecithin and gum arabic, have been applied to maintain the stability of the active ingredients during processing and storage. Furthermore, developments in personalized nutrition are prompting the creation of beverages targeted for specific metabolic profiles and genetic backgrounds (e.g., glucose response alteration and microbiome‐directed formulations). A schematic diagram of herbal concentrate juice production is illustrated in Figure 7, in which essential oils, fruit juice bases, and additives are mixed, homogenized, and concentrated under vacuum, followed by the formation of shelf‐stable functional beverages. This method is particularly advantageous for the development of energy and nutraceutical beverage concentrates containing potent extracts of herbal bioactive compounds.

### 3.4 Commercial Product Examples (International and Regional)

The global herbal beverage market has witnessed tremendous growth owing to increasing consumer preference for natural healthy products. These beverages provide hydration and contribute to physiological well‐being due to their content of antioxidants, anti‐inflammatory compounds, and adaptogens. The integration of traditional herbal wisdom and modern food technology has facilitated the commercialization of many functional drinks at the international and regional levels. Yogi Tea is one of the most recognizable herbal beverage brands, distributed extensively across the US and Europe. Their offerings feature specialty blends, like the “Bedtime,” “Detox” and “Throat Comfort” teas, which use ingredients like licorice root ( *Glycyrrhiza glabra* ), echinacea ( *Echinacea purpurea* ), and chamomile ( *Matricaria chamomilla* ) to target various health conditions like boosting immune defense, aiding digestion, or stress relief. These combinations are based on classical Ayurvedic principles and contemporary scientific evidence of the bioactive characteristics of polyphenols and flavonoids present in these herbs (Csatlos et al. 2023).

One more globally recognized herbal‐based beverage is GT's Kombucha (GT Dave's Kombucha), a fermented tea produced using a symbiotic culture of bacteria and yeast (SCOBY), typically including acetic acid bacteria (*Komagataeibacter* spp.) and yeasts (*Saccharomyces* spp. and *Zygosaccharomyces* spp.), and is known for its probiotic and antioxidant properties. The addition of botanicals such as ginger ( *Zingiber officinale* ), roselle ( *Hibiscus sabdariffa* ), and elderberry ( *Sambucus nigra* ) contributes to healthy gut and immune functions. According to Mihai et al. (2024), kombucha drinks have high phenolic content and radical scavenging activity, and they may be considered health‐promoting beverages. Red Bull Organics, a new market category developed under the Red Bull umbrella, distance itself from traditional energy drinks by removing artificial caffeine and adding botanic extracts (lemon balm, ginger, and cardamom) (Mihai et al. 2024). Herbal‐based energy drinks can also increase mental alertness and performance without the jitteriness typically experienced by synthetic stimulants (Jordan et al. 2023). Plant‐based enriched drinks with bioactive compounds have been established in the U.S. and Latin America. For example, it is compared U.S. and Peruvian commercial plant‐based functional beverages, and found that products made using ingredients such as açai, turmeric, and green tea had high concentrations of polyphenols, anthocyanins, carotenoids, and vitamin C. These bioactive compounds have antioxidant advantages and support the prevention of chronic diseases (De La Fuente‐Carmelino et al. 2025).

Tulsi green tea, prepared from holy basil ( *Ocimum tenuiflorum* L.), has been reported in India to act as an anti‐stress agent. *Holy basil* is also known in the Ayurvedic system for its immunomodulatory properties, with multiple studies supporting its antioxidative effects and blood glucose‐lowering potential (Junsi et al. 2025). Ready to Serve (RTS) herbal beverages have been the flavor of South Asia, particularly in India. Such drinks contain tulsi and pudina (*Mentha* spp.) or both along with tulsi, ginger, and mosambi (sweet orange), which are usually sold as summer coolers or immune boosters. RTS beverages with these herbs possess good consumer acceptability and sensory profile, which is attributed to their water‐soluble phytochemicals, such as menthol, gingerol, and eugenol, which are reported to have antibacterial and anti‐inflammatory activities (Biswas and Chowdhury 2019). The Herbal Mate Drink of South America uses *Ilex paraguariensis* (yerba mate). It is a popular drink and appreciated for its stimulant and hepatoprotective properties. It is shown that the high polyphenol content and antioxidant activity of yerba mate as well as its ability to modulate lipid metabolism and decrease liver damage (Lima et al. 2012). Regional medicinal plants are also used in the development of functional beverages in Southeast Asia. For example, it is formulated ginger‐lemongrass ( *Cymbopogon citratus* ) and stevia ( *Stevia rebaudiana* ) flavored herbal tea, which was found to possess strong antioxidant and α‐glucosidase inhibitory activity, suggesting potential anti‐diabetic effects. They are also valued for their organoleptic characteristics and have been suggested as potential commercial products for diabetes (Puspitojati et al. 2025). In China, commercial plant‐based functional beverages containing *Ampelopsis grossedentata*, *Pueraria lobata* , *Hovenia dulcis* , and *Artemisia annua* have the potential to control alcohol metabolism and improve liver function. It is reported that consumption of the beverage reduced blood alcohol and enhanced the activities of antioxidant enzymes (SOD and ALDH), which are involved in acute alcoholism and liver protection (Zhuo et al. 2025).

Similarly, in Romania, fermented soybean beverages fortified with *Chlorella vulgaris* have gained attention. This formulation, which is rich in microalgal peptides and antioxidants, supports gut health when combined with *Lactobacillus* strains. Csatlos et al. (2023) reported improved antioxidant capacity and probiotic viability, although post‐digestion bioavailability remains a challenge (Csatlos et al. 2023). In Ecuador, a fermented Andean blackberry ( *Rubus glaucus* ) beverage enriched with medicinal herbs, such as *Ocotea quixos* and *Amaranthus quitensis*, showed significant increases in total phenolics and antioxidant activity. Mihai et al. (2024) found that the addition of these herbs not only improved health metrics, but also enhanced flavor and consumer appeal (Mihai et al. 2024). Table 5 highlights various herbal‐based beverages across global markets, including tea, fermented drinks, and functional infusions. Products like Yogi Tea and GT's kombucha represent established brands, while newer innovations such as lemongrass–stevia tea (Indonesia) and chlorella‐fermented drinks (Romania) show regional development. The prices range from $0.50 to $4.99, reflecting the ingredient type and market scale.

**TABLE 5:** Overview of selected commercial herbal‐based beverages globally, highlighting drink type, geographic origin, manufacturers, production timeline, and approximate retail prices.

Drink name	Type	Location	Company	Manufacture year	Price (USD)
Yogi Tea (Bedtime, Detox, Throat Comfort)	Herbal tea	USA, Europe	Yogi Tea	1984–Present	$4.99/box
GT's Kombucha (Gingerade, Hibiscus, Elderberry)	Fermented drink	Global	GT's Living Foods	1995–Present	$3.50/bottle
Red Bull Organics	Energy drink (botanical)	Global	Red Bull GmbH	2018–Present	$2.99/can
Tulsi Green Tea	Herbal Tea	India	Organic India	2000–Present	$3.80/box
RTS Herbal Drinks (Tulsi, Mint, Mosambi)	Juice/Infused drink	India	Various Local Manufacturers	2015–Present	$0.50–$1.00/bottle
Herbal Mate Drink	Fermented/Probiotic tea	South America	Various Regional Brands	Traditional, Modern Since 2000s	$1.50/bottle
Chinese Herbal Detox Drink	Functional beverage	China	Local Pharm Brands	2010–Present	$1.20/bottle
Fermented Soybean + Chlorella Beverage	Probiotic fermented drink	Romania	University‐Linked Pilot Brand	2022–Present	$2.00/bottle (est.)
Andean Blackberry with Medicinal Herbs	Fermented herbal drink	Ecuador	Research/Start‐up Brands	2023–Present	$2.50/bottle
Herbal Lemongrass–Stevia Tea	Herbal infusion	Indonesia	University–Community Collab	2022–Present	$1.00/pack

## 4 Impact on Physicochemical and Functional Properties

The incorporation of herbal extracts into cold beverages significantly alters their physicochemical and functional properties. These changes are highly dependent on the type of herbal extract, beverage matrix (e.g., juice, flavored water, or fermented drink), and processing and storage conditions. Understanding these interactions is critical for designing stable, bioactive, and appealing beverages.

**FIGURE 8:**
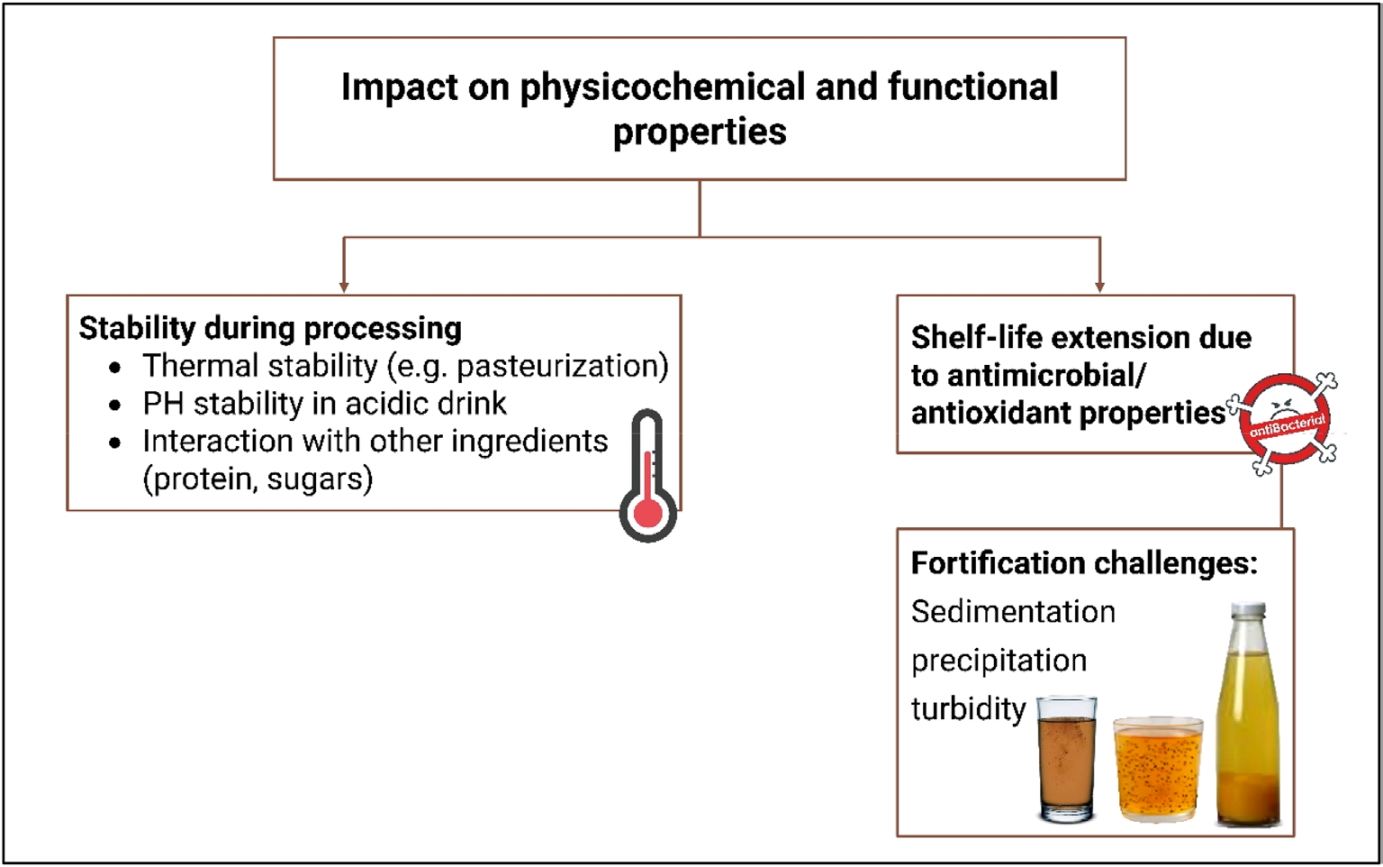
Impact of herbal extracts on physicochemical and functional properties of cold beverages.

Many herbal bioactives, such as polyphenols, flavonoids, and essential oils, are sensitive to heat, which poses challenges during thermal processing such as pasteurization. For instance, studies on *Hibiscus sabdariffa* and *Camellia sinensis* extracts have shown significant degradation of anthocyanins and catechins, respectively, at temperatures above 70°C (Preciado‐Saldaña et al. 2019). However, encapsulation technologies such as microencapsulation and nanoemulsion systems can enhance thermal protection. A study on turmeric‐based beverages has demonstrated that nano‐encapsulated curcumin retained 85% of its antioxidant activity after pasteurization compared to only 42% in the non‐encapsulated form. Many herbal beverages fall into the low‐pH range (3.0–4.5), which affects the chemical integrity of certain bioactive compounds. Polyphenols, such as epigallocatechin gallate (EGCG), in green tea are known to degrade rapidly under acidic conditions (Patel et al. 2023). A 50% decrease in the TPC of kombucha‐based herbal beverages is reported after 14 days of storage at low pH due to hydrolytic and oxidative degradation (Mihai et al. 2024). However, certain compounds such as anthocyanins from berries remain stable in acidic environments and even enhance beverage coloration (Almeida et al. 2023). Moreover, interactions between herbal bioactives and beverage components such as proteins and carbohydrates can alter both functional efficacy and sensory quality. For example, polyphenols can bind to milk proteins in smoothies, reducing their bioavailability and improving their antioxidant stability (Wang et al. 2021). Fiber‐rich plant‐based beverages with added herbs may experience altered viscosity, which affects mouthfeel and suspension stability. In one study, a synbiotic drink containing chicory inulin and *Lactobacillus plantarum* with green tea extract showed a noticeable increase in viscosity and sedimentation compared to the controls (Zhang et al. 2023). Additionally for shelf‐life extension due to the antimicrobial and antioxidant properties of herbal extracts significantly contribute to shelf‐life extension by inhibiting microbial growth and oxidative deterioration. In another similar study, the shelf life of pomegranate juice was extended from 5 to 15 days during refrigeration when clove ( *Syzygium aromaticum* ) and cinnamon ( *Cinnamomum zeylanicum* ) oils inhibited the growth of *E. coli* and *S. aureus* (Iseppi et al. 2024). Similarly, an extract of basil ( *Ocimum basilicum* ) in lemon juice resulted in a 40% reduction in the microbial count on the stored dates after 10 days compared with the untreated treatment (Naher et al. 2023). Herbal antioxidants, including rosmarinic acid (from rosemary) and ellagic acid (from pomegranate peel), are used to limit lipid oxidation and other discoloration in fat‐containing smoothies or nut‐based milk beverages. For example, a rosemary extract‐enriched chia smoothie retained 85% of its DPPH antioxidant activity after 30 days of cold storage at 4°C (Al‐jaafreh 2024; Martínez et al. 2019). Despite their benefits, herbal extracts often pose formulation challenges, and sedimentation is a major concern in beverages containing insoluble or poorly soluble herbal components such as turmeric or ginger powders. Turmeric‐fortified smoothies showed up to 60% phase separation after 3 days, unless stabilizers such as pectin or xanthan gum were added (Jacob et al. 2024). Polyphenol–protein and polyphenol–mineral interactions can result in visible precipitation or haze, which negatively impacts the product's visual appeal. Green tea extract added to calcium‐fortified almond milk formed visible flocculates within 48 h of storage owing to polyphenol–calcium complexation (Qin et al. 2023). High turbidity levels are common in herbal‐infused drinks because of the presence of suspended particles or emulsions. Controlled homogenization and the use of stabilizing emulsifiers (e.g., gum arabic and lecithin) are necessary to maintain dispersion. A case study of a herbal‐fortified fermented soy drink showed turbidity values exceeding 150 NTU without emulsifiers, but with proper stabilization, values remained below 40 NTU over 10 days (Csatlos et al. 2023). Figure 8 summarizes the influence of the herbal extracts on the physicochemical and functional properties of the fortified cold beverages. These effects include thermal and pH stability, interaction with proteins and sugars, enhancement of shelf life through antimicrobial and antioxidant actions, and formulation challenges, such as sedimentation, precipitation, and turbidity. Table 6 presents an integrated overview of the effects of the herbal extracts on the physicochemical and functional qualities of the fortified fruit juices. It covers key aspects such as pH‐dependent phytochemical stability; heat sensitivity of bioactive compounds such as curcumin and catechins; and compatibility with beverage ingredients such as proteins, sugars, and fibers. Additionally, the table outlines how herbal additives extend shelf life through antioxidant and antimicrobial actions and lists common fortification challenges, such as sedimentation and turbidity, along with suggested formulation solutions.

**TABLE 6:** Effects of herbal extracts on the physicochemical and functional properties of fruit juices: this table summarizes how various herbal additives impact pH stability, thermal properties, ingredient interactions, shelf life, and fortification issues from different fruit juice matrices.

Fruit type	Herb type	pH range	Improvement	Aim	References
Orange juice	Green tea	3.2–4.0	Improved stability of catechins	Antioxidant protection	Pathania and Dubey (2025)
Apple juice	Ginger extract	3.5–4.2	Retention of bioactives	Anti‐inflammatory properties	Hassanzadeh et al. (2023)
Pomegranate juice	Hibiscus	2.8–3.5	Stabilized anthocyanins	Color retention	Fischer et al. (2013)
Mango juice	Lemongrass	3.6–4.5	Reduced degradation of citral	Flavor preservation	Nayana and Wani (2024)
Grape juice	Chamomile	3.0–3.6	Better preservation of apigenin	Calming effects	Hostetler et al. (2013)

Thermal stability of herbal juices					
Juice type	Herb type	Temp range (°C)	Phytochemical loss/Retention	Improvement	References
Lemon juice	Mint	70°C–90°C	Moderate loss of menthol	Flavor enhancement	Yetisen et al. (2022)
Carrot juice	Turmeric	80°C–95°C	Curcumin degradation at > 85°C	Anti‐inflammatory boost	Pathania and Dubey (2025)
Apple juice	Cinnamon	65°C–85°C	Preserved cinnamaldehyde at < 80°C	Stability improvement	Nayana and Wani (2024)
Beet juice	Ginger	70°C–90°C	Partial loss of gingerols	Antioxidant enhancement	Pavlović et al. (2022)
Mixed berry juice	Green Tea	60°C–80°C	EGCG stable below 75°C	Antioxidant enrichment	Radeva‐Ilieva et al. (2025)

Ingredient interactions with herbal juices					
Juice type	Interacting ingredient	Herb type	Interaction type	Observation	References
Orange juice	Protein (Whey)	Green Tea	Slight turbidity	Protein–polyphenol interaction	Lermen et al. (2024)
Berry juice	Sugar (Glucose)	Cinnamon	Flavor masking	Sugar–volatile interactions	Mavadiya et al. (2025)
Apple juice	Fiber (Inulin)	Hibiscus	Stable dispersion	Textural enhancement	Pathania and Dubey (2025)
Pineapple juice	Vitamin C	Turmeric	Degradation of curcumin	Oxidative instability	Nayana and Wani (2024)
Grape juice	Milk protein	Chamomile	Protein–flavonoid complex	Reduced bioavailability	Lermen et al. (2024)

Shelf‐life extension in herbal juices				
Juice type	Herb type	Shelf life duration	Improvement	References
Pomegranate juice	Cinnamon	30 days	Antioxidant preservation	Nayana and Wani (2024)
Mixed fruit juice	Green Tea	45 days	Reduction in microbial load	Pathania and Dubey (2025)
Beetroot juice	Ginger	35 days	Color and flavor retention	Junsi et al. (2025)
Carrot juice	Lemongrass	28 days	Enhanced freshness	Szwajgier et al. (2022)
Orange juice	Hibiscus	30 days	Improved phenolic stability	Romeo et al. (2020)

Fortification challenges in herbal juices				
Juice type	Herb type	Challenge observed	Suggested solution	References
Pineapple juice	Moringa	Sedimentation of protein fractions	Reduce concentration or encapsulate	Pathania and Dubey (2025)
Carrot juice	Turmeric	Curcumin precipitation	Use nanoemulsion for delivery	Nayana and Wani (2024)
Grape juice	Cinnamon	Turbidity from essential oils	Use emulsifiers or clarifying agents	Mavadiya et al. (2025)
Beet juice	Green Tea	Precipitate formation	Chelate stabilizers may help	Szwajgier et al. (2022)
Lemon juice	Ginger	Layer separation	Optimize homogenization conditions	Moskowitz (2002)

## 5 Sensory Characteristics and Consumer Acceptance

The incorporation of herbal extracts in beverages has a major impact on sensory characteristics such as taste, aroma, color, and mouth feel, and thus the acceptability of the product by the consumer. Polyphenol‐enriched herbs, such as green tea, hibiscus, and turmeric, can impart beneficial health properties, but at the expense of bitterness and astringency, which confront consumers on palatability. For instance, catechins in green tea can boost antioxidant potential, although they also contribute to a characteristic bitterness that might not be liked by all consumers (Pathania and Dubey 2025). Hibiscus, on the other hand, adds a red color due to anthocyanins that can improve color appeal and possibly affect perceived sourness (Junsi et al. 2025). In contrast, lemongrass and mint have more acceptable flavor profiles and are often used to mask the unpleasant taste of polyherbal formulations (Nayana and Wani 2024). It is reported that the addition of herbal infusions to fruit juices may alter mouthfeel due to the presence of polysaccharides and saponins that may increase viscosity or generate a slimy sensation (Hussain et al. 2015). For instance, *Aloe vera* and moringa extracts exhibited enhanced thickness in fortified mango and orange juices, and thus influenced the drinkability and texture in general (Abushal et al. 2024; Lermen et al. 2024). Hibiscus‐fortified beverages were tested hedonically, and subjects found the color and potential health benefits favorable, whereas the sour and metallic aftertaste was less favored, indicating a need for flavor‐adjustment agents (Paloukopoulou et al. 2024). Sweeteners and flavor‐masking agents have been found to have a substantial impact on sensory acceptability, as documented in the literature. Moreover, natural low‐calorie sweeteners, such as stevia, monk fruit, as well as fermentation‐derived erythritol, have been effectively employed to reduce bitterness and improve the taste profile of green tea and bitter melon‐based peptide beverages without contributing to the caloric burden (Desva et al. 2023; Mavadiya et al. 2025). Moreover, citrus‐based essential oils or tropical fruit flavorings can be used to cover the earthy or medicinal aftertaste of adaptogenic herbs, including ashwagandha and ginseng (Tang et al. 2023). Color impacts consumer acceptance as well, where the deep red derived from hibiscus anthocyanins is repeatedly associated with perceived naturalness and healthiness and extends acceptance among health‐minded populations (Kang et al. 2023). Moreover, consumer choice experiments, for example, show that familiarity with specific herbs enhances acceptance (Mathew et al. 2022). Beverages containing locally known herbs such as mint or ginger tend to score better on hedonic testing than products formulated with less familiar ingredients such as holy basil or spirulina, which might require a more educated or niche consumer base. Texture‐modifying approaches have also proven to be effective. For instance, the addition of microencapsulated herb extracts or use of hydrocolloids, such as pectin or guar gum, can lead to uniformity and minimize variations in mouth feel (Zhou et al. 2024).

**FIGURE 9:**
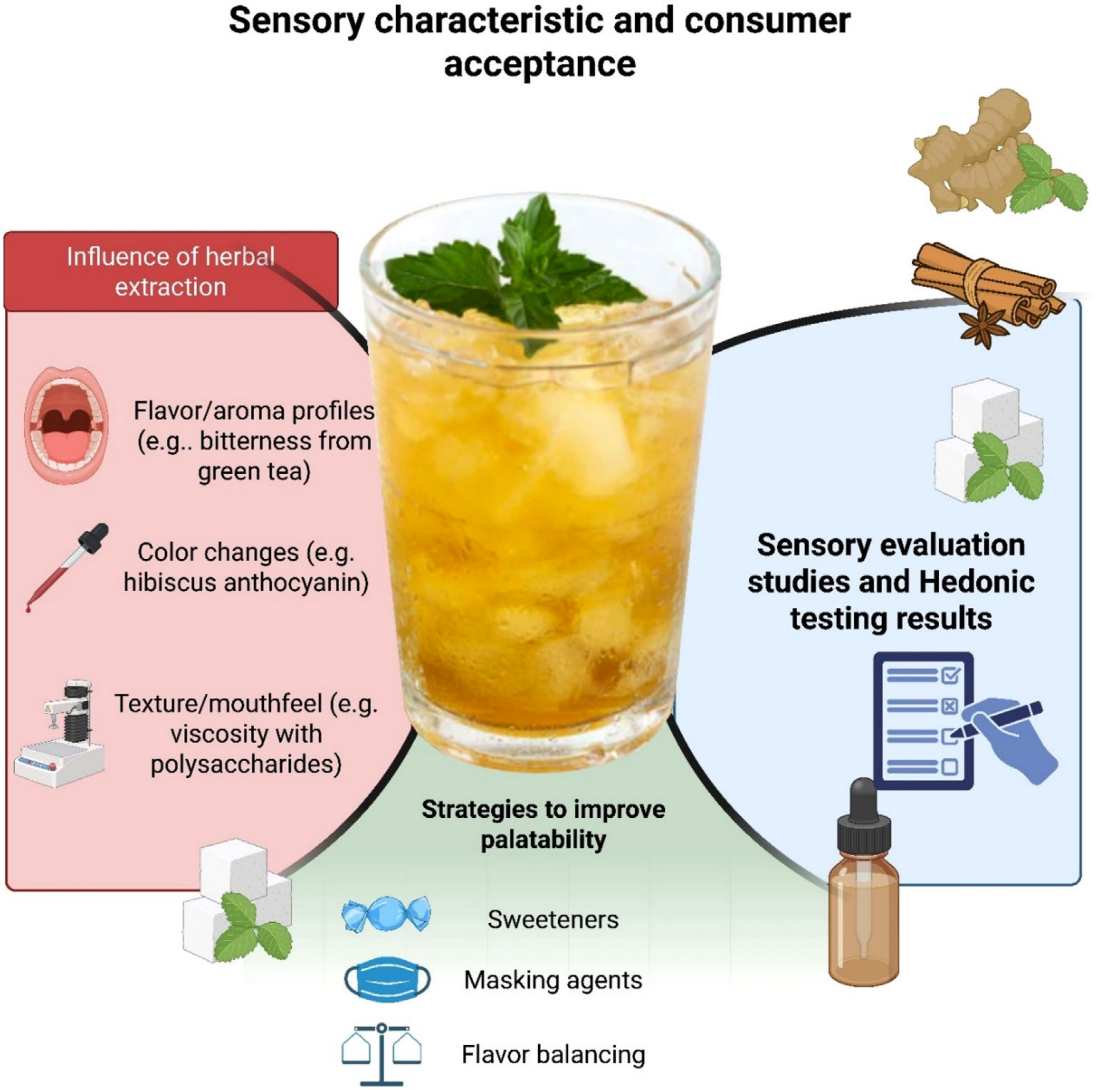
Influence of herbal extracts on sensory characteristics and strategies for improving consumer acceptance.

Sensory preferences also differ greatly according to age and regional taste preferences. A cross‐sectional study by Martínez‐Subirà et al. (2025) from a variety of countries demonstrated that the older some consumers are, the less strongly herbal notes are appreciated. In contrast, the younger the consumer, the more adventurous they may be, often preferring bolder, more functional notes. This variability emphasizes the need to customize herbal beverage formulations depending on the target market segment (Martínez‐Subirà et al. 2025). Furthermore, it is described that promoting health and education campaigns enhances consumer tolerance to odd flavors when they are associated with actual health benefits (Tuorila and Hartmann 2020). General and specific aspects of herbal extracts in drinks: The use of herbal extracts in quality drinks offers possibilities, as well as special sensory influences on beverages. Satisfying consumer acceptance by maximum formulation by masking agents, sweeteners, and flavor carriers based on what the consumer already knows and informs the consumer about the health attitude. Scientific evaluation, along with consumer sensory testing, provides a rational strategic formulation approach that helps product developers optimize the development of palatable functional herbal beverages. Figure 9 shows the strong contribution of herbal extracts to the sensory attributes of fortified beverages (taste, color, and mouthfeel).

## 6 Health Benefits and Scientific Evidence

**FIGURE 10:**
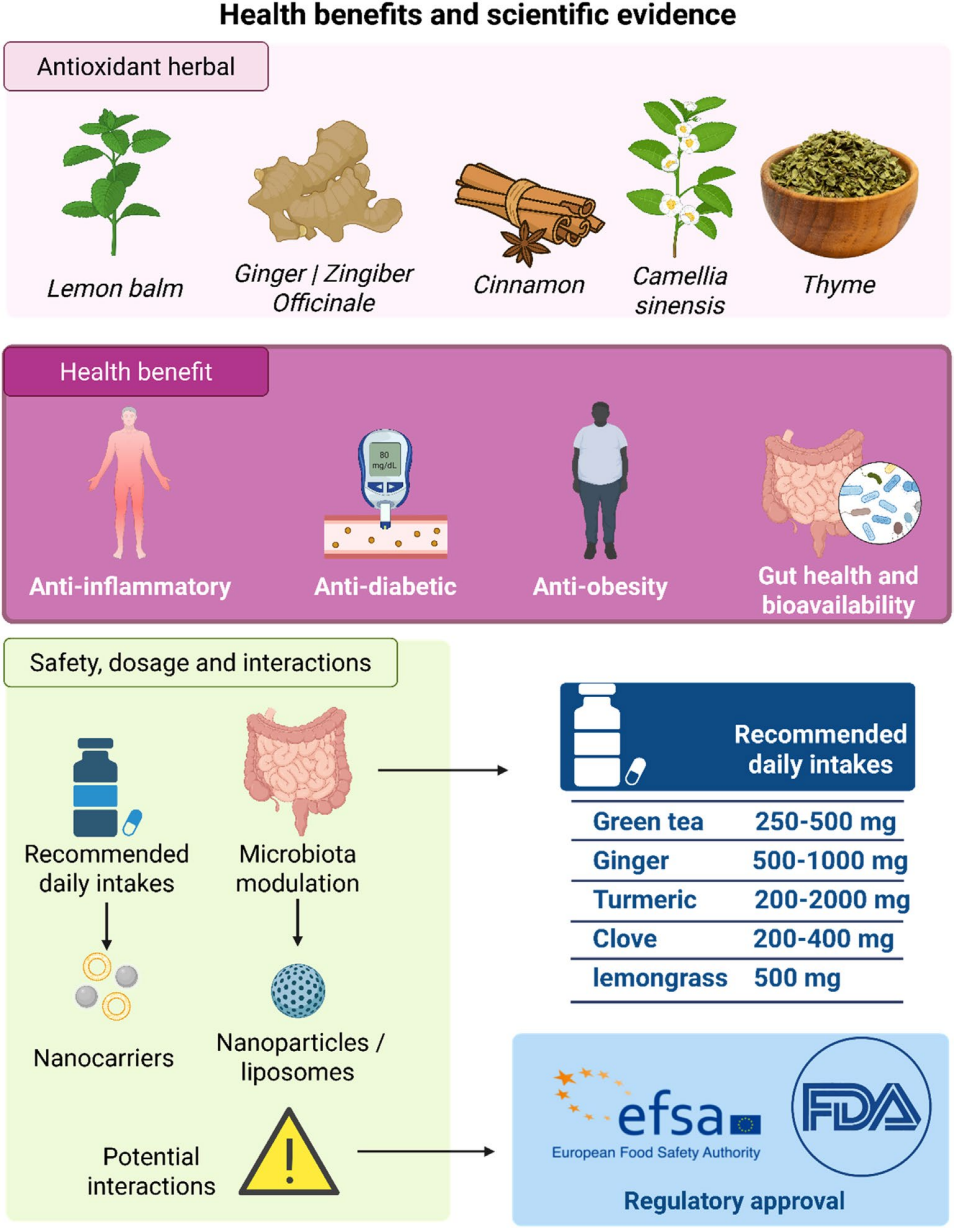
Overview of key antioxidant herbs, associated health benefits, safety considerations, and regulatory guidelines.

The health benefits of herbal extracts used in beverages have been extensively studied in both the clinical and preclinical contexts. These benefits are largely attributed to the rich profiles of polyphenols, flavonoids, anthocyanins, and other secondary metabolites with proven biological activities. Among the most widely studied herbs are green tea ( *Camellia sinensis* ), ginger ( *Zingiber officinale* ), turmeric ( *Curcuma longa* ), hibiscus ( *Hibiscus sabdariffa* ), cinnamon (*Cinnamomum* spp.), and ginseng ( *Panax ginseng* ). Each of these botanicals has distinct biofunctional effects, including antioxidative, anti‐inflammatory, metabolic, and gut health modulation, alongside measurable effects in standardized assays and human studies. Standard antioxidant assays, such as DPPH, ABTS, and FRAP, have demonstrated the significant radical‐scavenging capacities of herbal extracts. Green tea catechins, particularly epigallocatechin gallate (EGCG), are consistently used in these assays. It is analyzed that green tea extract exceeded 2.0 mmol Fe^2+^/g dry extract, outperforming both black tea and turmeric (Pathania and Dubey 2025). Table 7 provides a comprehensive overview of the recommended daily doses and safety considerations associated with various herbal ingredients commonly used in beverage fortification. Hibiscus anthocyanins were also found to have a high antioxidant potential in DPPH and ABTS assays, with IC₅₀ values ranging from 11 to 25 μg/mL (Junsi et al. 2025). Similar strong performance was reported for cinnamon bark extracts, with cinnamaldehyde contributing significantly to FRAP values in ethanolic extracts (Mavadiya et al. 2025). Herbal extracts have demonstrated robust anti‐inflammatory activities via various pathways. Curcumin inhibits NF‐κB and COX‐2 expression in both murine and human models. A meta‐analysis by Abushal et al. (2024) of 12 clinical trials found a significant reduction in C‐reactive protein (CRP) and interleukin‐6 (IL‐6) levels in patients taking turmeric extract over 6–12 weeks. Gingerols in ginger suppress TNF‐α and IL‐1β, with clinical evidence from osteoarthritis trials showing improved pain and mobility after 8 weeks of supplementation (Lermen et al. 2024). For anti‐diabetic potential, Cinnamon polyphenols have been shown to improve fasting glucose levels and insulin sensitivity. In a 12‐week double‐blind randomized controlled trial (RCT) involving 120 patients with type 2 diabetes, cinnamon supplementation (3 g/day) significantly decreased HbA1c and fasting blood glucose (Desva et al. 2023). Green tea also showed improved insulin function and glucose uptake in animal models and humans, which was attributed to the effect of catechins on AMPK activation. A number of herbal bioactives have been linked with health‐promoting effects against metabolism‐related endpoints via complementary pathways. Ginsenosides from ginseng have an effect on glucose homeostasis and pancreatic β‐cell protection, and polyphenol‐enriched herbs (green tea, cinnamon, hibiscus) are associated with weight management and lipid metabolism benefits. The green tea catechins have been reported to play a role in stimulating thermogenesis and fat oxidation, cinnamon supplementation has a beneficial effect on glycemia control, and hibiscus polyphenols influence lipid metabolism pathways leading to reduced LDL cholesterol and triglyceride levels in human studies (Mathew et al. 2022). Moreover, the herbal extracts demonstrated robust anti‐inflammatory activity via various pathways. Curcumin inhibits NF‐κB and COX‐2 expression in both murine and human models. A meta‐analysis by Abushal et al. (2024) covering 12 clinical trials found a significant reduction in C‐reactive protein (CRP) and interleukin‐6 (IL‐6) levels in patients taking turmeric extract over 6–12 weeks (Abushal et al. 2024). Gingerols in ginger suppress TNF‐α and IL‐1β, with clinical evidence from osteoarthritis trials showing improved pain and mobility after 8 weeks of supplementation (Lermen et al. 2024). For anti‐diabetic potential, Cinnamon polyphenols have been shown to improve fasting glucose levels and insulin sensitivity. In a 12‐week double‐blind RCT involving 120 patients with type 2 diabetes, cinnamon supplementation (3 g/day) significantly decreased HbA1c and fasting blood glucose (Desva et al. 2023). Green tea catechins increase thermogenesis and fat oxidation; a study by Mathew et al. (2022) showed a mean body weight reduction of 1.2 kg in overweight subjects after 3 months of consumption. Hibiscus polyphenols reduce lipid accumulation by modulating lipid metabolism genes, and are associated with improved LDL and triglyceride profiles in human trials. Gut microbiota modulation has emerged as a vital mechanism through which herbal extracts exert health effects. Ginseng, turmeric, and green tea have been found to favorably alter the gut microbiota by promoting beneficial genera, such as *Bifidobacterium* and *Lactobacillus*, while reducing pathogenic species (Mathew et al. 2022). A pilot study demonstrated an increase short‐chain fatty acid production and gut epithelial integrity in mice supplemented with ginseng tea. Nevertheless, these positive outcomes are limited by the low bioavailability of some phytochemicals such as curcumin and EGCG (Kang et al. 2023). Nanoparticle encapsulation, liposomal systems, and bioenhancers such as piperine have been investigated to solve these problems with positive results (Kumar et al. 2022). Lastly, although many of these health effects are evidence‐based, translating them into legally valid functional claims continues to be problematic. However, the European Food Safety Authority (EFSA) has only approved no more than a small number of claims so far, notably including “green tea contributes to the protection of DNA from oxidative damage,” but has also refused a number of others because of the absence of evidence in humans or because of bioavailability. In contrast, the U.S. Food and Drug Administration (FDA) permits structure–function claims under the Dietary Supplement Health and Education Act (DSHEA), allowing labels such as “supports cardiovascular health,” but prohibits disease‐specific claims without pre‐market approval. For instance, while turmeric is recognized for its anti‐inflammatory potential, products cannot claim “treats arthritis” unless validated in clinical trials and approved as a drug. Safety, dosage, safety profiles, and recommended dosages are crucial when formulating herbal beverages. Most clinical studies have used standardized extracts with clear dosage limits. For green tea catechins, a common safe dose is up to 500 mg/day (EFSA et al. 2023), whereas for curcumin, clinical safety is documented up to 2000 mg/day in divided doses with few minor adverse effects reported, such as gastrointestinal upset. In particular, multiple human studies found curcumin safe in doses ranging from 1125 to 2500 mg per day without significant toxicity (Chainani‐Wu 2003). Ginseng is generally safe at 200–400 mg/day, but higher doses may induce insomnia or headaches (Reay et al. 2005). Hibiscus extract is safe up to 500 mg/day, but may significantly lower blood pressure, cautioning against concurrent use with antihypertensive drugs (Almajid et al. 2023). Interactions with pharmaceuticals are of major concern. Ginseng may interfere with warfarin and other anticoagulants by altering the CYP450 metabolism. Similarly, caffeine‐containing herbs, such as green tea, may elevate blood pressure or interact with beta blockers. It is essential to consider such interactions during formulation and marketing, particularly in populations with chronic diseases or polypharmacy. The EFSA and FDA guidelines recommend warning labels where interactions are suspected. As depicted in Figure 10, several antioxidant‐rich herbal extracts, including ginger, green tea, turmeric, and cinnamon, have been associated with a wide range of health‐promoting effects, including anti‐inflammatory, anti‐diabetic, and anti‐obesity effects, as well as improved gut health and nutrient bioavailability. These effects are linked to the rich contents of polyphenols, flavonoids, and other bioactive compounds. Safe consumption is guided by regulatory bodies such as EFSA and the FDA, which provide recommended intake ranges, whereas novel delivery systems such as nanocarriers and liposomes enhance absorption and reduce interaction risks.

**TABLE 7:** Extended herbal beverage dosage and safety table. This table summarizes the recommended daily doses, potential safety concerns, and upper intake limits of commonly used herbal ingredients in functional beverages.

Herbal ingredient	Beverage type	Recommended daily dose	Upper limit/Warning	References
Green tea extract	Tea, juice	250–500 mg catechins	800 mg/day liver risk	Pathania and Dubey (2025)
Hibiscus	Juice, Tea	200–500 mg extract	Avoid excess in hypotension	Junsi et al. (2025) and Kang et al. (2023)
Ginger	Tea, Juice	500–1000 mg extract	Caution in gallstones	Lermen et al. (2024)
Turmeric (Curcumin)	Functional drink, Tea	500–2000 mg curcumin	GI upset in high doses	Abushal et al. (2024)
Cinnamon	Tea, Juice	1–3 g powder	Liver damage in excess	Desva et al. (2023) and Kumar et al. (2022)
Lemongrass	Tea	300–500 mg	Safe in moderation	Nayana and Wani (2024)
Mint	Tea, Juice	300–600 mg	Generally safe	Tang et al. (2023)
Chamomile	Tea	200–400 mg	Drowsiness possible	Mathew et al. (2022)
Basil	Tea	300–600 mg	Safe short‐term	Tang et al. (2023)
Holy Basil (Tulsi)	Tea	300–500 mg	May lower blood sugar	Kumar et al. (2022)
Moringa	Juice, Tea	1–2 g powder	Avoid in pregnancy	Paloukopoulou et al. (2024)
*Aloe vera*	Juice	100–300 mg	Laxative effect in high dose	Desva et al. (2023)
Fennel	Tea	250–500 mg	Hormonal effects	Abushal et al. (2024)
Cardamom	Tea	500–1000 mg	GI upset in high doses	Nayana and Wani (2024)
Clove	Tea	200–400 mg	May irritate GI tract	Zhou et al. (2024)
Garlic extract	Juice	300–900 mg	Bleeding risk	Authority (2023)
Black pepper	Tea	100–300 mg	Enhances drug absorption	Lermen et al. (2024)
Fenugreek	Tea	500–1000 mg	Hypoglycemia risk	Kang et al. (2023)
Nettle leaf	Tea	200–400 mg	Diuretic effects	Mathew et al. (2022)
Lavender	Tea	200–400 mg	Safe in small doses	Pathania and Dubey (2025)
Peppermint	Tea	200–400 mg	Mild GI effects	Mavadiya et al. (2025)
Dandelion root	Tea	500–1000 mg	Diuretic	Paloukopoulou et al. (2024)

## 7 Emerging Trends and Future Directions

**FIGURE 11:**
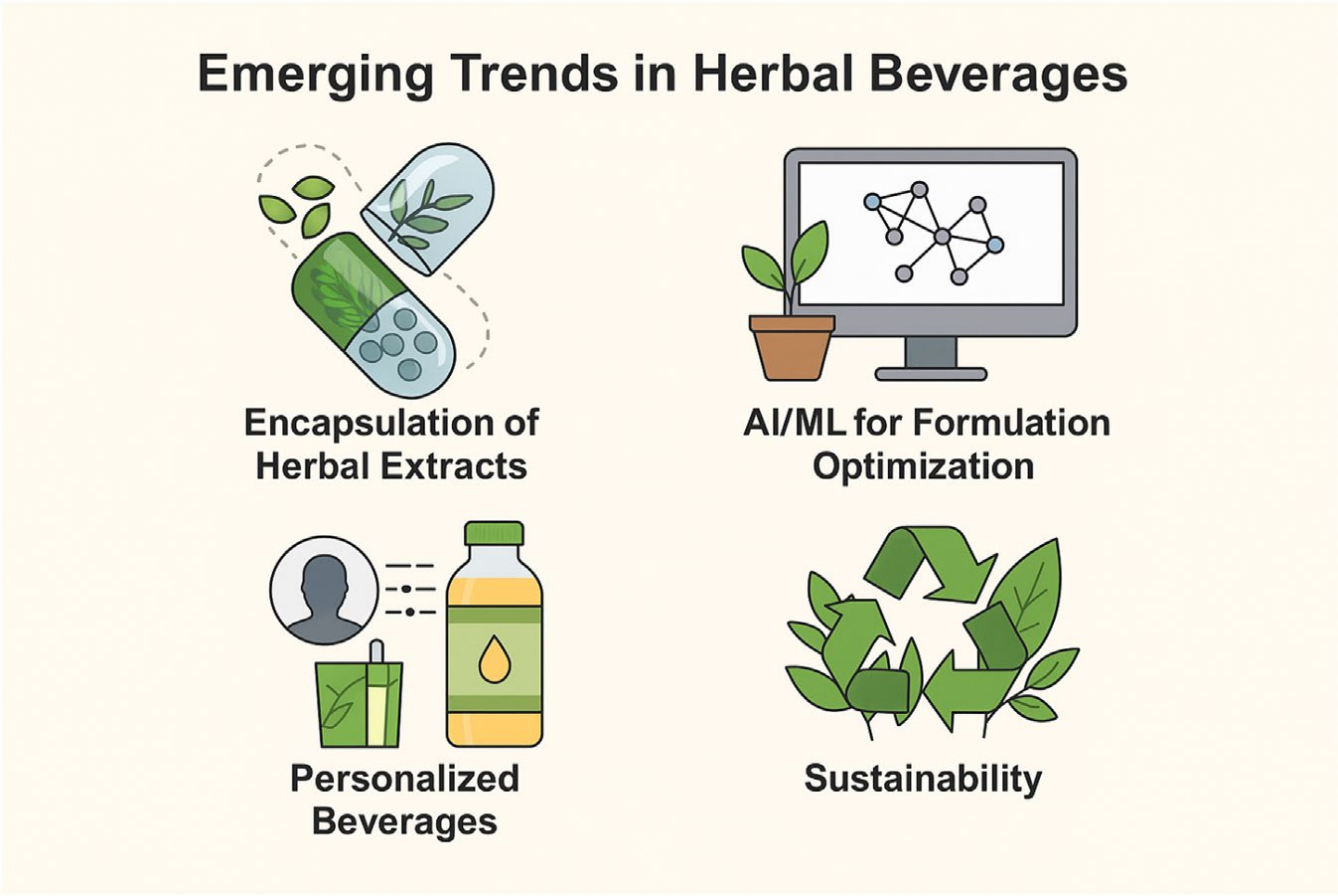
Emerging trends in herbal beverages. Key Innovations are encapsulation, leveraging AI/ML for optimizing formulations, personalized beverages via nutrigenomics, and sustainability.

Innovative trends in herbal beverages are due to advances in science coupled with increasing consumer health consciousness, personalization, and sustainability. One significant development has been the encapsulation of herbal extracts that enhances the stability and bioavailability of labile bioactive compounds such as curcumin, catechins, and anthocyanins. These compounds can be protected from degradation during processing and can be targeted for release into the body by methods such as nanoencapsulation and liposomes. Artificial intelligence (AI) and machine learning (ML) are increasingly used to aid formulation optimization by predicting the stability, sensory properties, and synergistic effects of herbal combinations. In addition, these constituents accelerate the detection of new bioactive compounds in plant extracts. Individualized herbal infusions, which are designed according to age, gender, and health, are commonly available and promoted by the emerging discipline of nutrigenomics and knowledge about the microbiome. Smart dispensers and subscription services make it easy to customize on the fly. Thus, sustainable development is also important. This encompasses ethical herb procurement, a decrease in synthetic additives, green extraction technologies, and upcycling of herbal residues. How data, personalization, and the environment will change innovation forever; the latest and future developments in herbal beverages are shown in Figure 11. Such solutions range from innovative delivery systems as encapsulation technologies for bioactive protection and delivery, and AI/machine learning‐driven formulation design to personalized health‐based beverage solutions and a sustainable focus on environmental practices.

## 8 Conclusion

Traditional herbal drinks have an appealing tradition and science synergy, and have been used clinically and preclinically for various health benefits. The high content of polyphenols, flavonoids, and other bioactive compounds leads to antioxidant, anti‐inflammatory, antidiabetic, and gut‐modulating effects. However, technological advancements, such as encapsulation, AI‐assisted formulation, and personalized nutrition platforms, are improving the efficacy, stability, and consumer appeal of next‐generation products. However, several challenges remain. Sensory components, such as bitterness, color degradation, precipitation, and nutritional fortification and regulation constraints, still affect product development. In addition, the variability in herb composition, poor bioavailability of some compounds, and lack of large‐scale clinical evidence impede the general acceptance of therapeutic nutrition. Further progress will have to build on such multidisciplinary approaches that combine food technology, regulatory science, and consumer education. Continued development of clinical trials, mechanistic studies, and sustainable procurement practices will reinforce the veracity and utility of herbal beverages. With the further merging of food and health systems, herbal beverages have the potential to play a central role in preventive health, functional food design, and integrative care.

## Author Contributions

**Tarek Gamal Abedelmaksoud:** writing – review and editing, investigation, data curation, formal analysis, resources, validation. **Rawaa H. Tlay:** writing – original draft, writing – review and editing, resources, investigation, formal analysis, validation. **Ammar B. Altemimi:** resources, validation, writing – review and editing. **Mohammed N. Saeed:** resources, validation, writing – review and editing, writing – original draft. **Syamand Ahmed Qadir:** writing – review and editing, methodology, data curation. **Farhang Hameed Awlqadr:** conceptualization, writing – original draft, writing – review and editing, project administration, visualization, supervision. **Othman Abdulrahman Mohammed:** validation, resources.

## Funding

The authors have nothing to report.

## Conflicts of Interest

The authors declare no conflicts of interest.

## Data Availability

The data that support the findings of this study are available from the corresponding author upon reasonable request.
